# Membrane insertion and dimerization of glycophorin-A mutations studied by free energy simulations

**DOI:** 10.1016/j.bpj.2025.12.039

**Published:** 2026-01-05

**Authors:** Cong Van Quy, Martin Kulke, Martin Zacharias

**Affiliations:** 1 Physics Department and Center of Protein Assemblies, Technical University of Munich, Garching, Germany; 2 Computational Biomedicine, Forschungszentrum Jülich, Jülich, Germany

## Abstract

Numerous proteins are associated with cellular membranes and often contain single or multiple membrane-spanning helices. These helices can mediate membrane protein interactions to form functional complexes involved in enzymatic or signaling processes. A detailed understanding of interactions and driving forces is essential for the understanding of membrane protein association and for membrane protein complex design. The glycophorin-A transmembrane helical dimer has been studied extensively by biochemical and structural methods, including mutagenesis of dimer interface residues. We use alchemical free energy simulations to investigate the effect of amino acid substitutions on the binding free energy and the change in membrane insertion free energy. Simulations on more than 30 substitutions were performed in different lipid environments and resulted in overall good agreement with experimental data, both for the change in membrane insertion and dimerization free energies. For the membrane insertion, the simulations slightly underestimated the stabilization due to larger nonpolar residues and overestimated the destabilization by substitution with polar residues. Interestingly, very little influence of the lipid type on changes in membrane insertion free energy was observed. The influence of lipid environment on the calculated binding free energy changes was also modest for most substitutions but significant for mutations that affect the glycine residues in the central GxxxG interaction motif. Enhanced lipid dynamics of unsaturated lipids may compensate for conformational changes in the case of mutations of interface glycine to larger residues. Mutations, especially of residues V84 and T87 to other polar and nonpolar residues, allowed us to estimate the contribution of additional hydrogen bonds (∼−1.0 kcal/mol) and removal of methyl groups (∼0.5 kcal/mol) to dimerization. Our study also demonstrates the usefulness of alchemical free energy simulations to quantify the influence of amino acid substitutions on membrane helix association and could be valuable for the design of new membrane protein interactions.

## Significance

Many membrane proteins consist of several membrane-spanning helices or are membrane anchored by single helices that can mediate dimer or oligomer formation. A detailed understanding of the driving forces and residue-specific affinity of helix association and how it depends on the membrane lipids is still missing. In our study, we use alchemical free energy simulations to investigate the effect of various mutations in the glycophorin-A transmembrane helical segment on insertion and dimerization. The results are in overall good agreement with experiment and offer an improved understanding how amino acid substitutions can affect helix insertion and association in membranes at molecular detail. The approach also holds the potential for applications to more complex membrane proteins.

## Introduction

Transmembrane (TM) proteins play pivotal roles in various cellular processes. Among the diverse classes of TM proteins, *α*-helix is the dominant architecture. In the context of the membrane protein assembly, the driving forces of transmembrane helix assembly and recognition remain conundrums (1,2,3) due to their meticulous intertwining with highly complex surrounding lipid environments (4,5,6). In particular, either enhancing or reducing the association degree could lead to dysfunction of the membrane protein complex, implicating concerning diseases such as cancer, heart disease, Alzheimer disease, and cystic fibrosis, etc. (5,7). Serving as the first step to high-order folding, such as oligomerization, TM protein dimerization presents the simplest model to investigate the structures and energetics of TM proteins in general.

Despite extensive studies over the last decades, the mechanisms of membrane protein assembly and stability, as well as the apparent existence of highly polar residues in some proteins in the hydrophobic membrane core, remain elusive (2,8,9). Several challenges lead to ambiguities in interpretation, including varying experimental conditions (10,11), the use of a simplified monomer-dimer model, neglected relevant equilibria (6,12), quantitative measurement based on protein concentration (6,13), or system selection restricted to known cases (14,15,16). An essential milestone has been dedicated to the belief that the lateral interaction is primarily driven by the sequence motif of simple recognizable amino acids of the *α*-helical interface (10,17,18,19), supporting the understanding of the strength and specificity of TM helix dimerization (14,15,16).

A famous example is the GxxxG motif found in the membrane-helical part of glycophorin-A (GpA) (10,14). GpA is a highly glycosylated integral transmembrane protein of the erythrocyte membrane. Its single membrane-spanning helix can dimerize in the membrane with a GxxxG motif forming the central contact region. Nonetheless, the sequence motif concept cannot fully describe the driving force of membrane protein association due to the existence of diverse motifs of other stable helical structures. For instance, the GxxxG motif in GpA was shown to be neither necessary nor sufficient for helical dimerization (10,15,19). Several assays, such as TOXCAT (20,21), GALLEX (12), and dsT *β* L (6), have progressively measured the thermodynamics of insertion and stability of the GpA dimer indicating that not only specific protein-protein interactions but also nonspecific forces such as lipid-lipid, lipid-protein contacts (9,10,22,23), or even interplay between protein and other diffusive external molecules (24,25) can influence affinity and specificity.

Molecular dynamics (MD) simulations offer atomistic insight into membrane protein interactions. In order to reduce the computational costs, coarse-grained simulations have been widely used to investigate helix-helix association (22,26,27,28,29). However, a recent report by Mahraryta et al. (11) confirms the necessity of an atomic resolution representation and a rigorous geometrical framework in accurately calculating the potential of mean force (PMF) of the TM helix association. Therefore, such PMF calculations are computationally expensive and not practical for high-throughput screening of mutations.

Alternatively, alchemical simulations can be used to study the effect of mutations on membrane insertion and interaction, avoiding PMF calculations along large spatial displacements (30,31,32). Initial alchemical simulations on the GpA dimer employed a simple dodecane single layer as lipid environment (1,33), neglecting the important role of natural membrane architecture on the dimerization propensity (10,20,34,35). Alchemical protocols have also been applied to membrane proteins interacting with small drug-like ligands (10), but so far not to systematically study the effect of mutations on membrane protein association.

As the main focus, we present an atomistic alchemical simulation protocol to quantify the thermodynamics of TM helix partitioning and dimerization upon mutation and examine how these properties are modulated by lipid environment. We use GpA as a model system since it allows a direct comparison to experimental data based on different experimental methods. For a diverse set of 38 GpA mutations, we find good agreement of calculated changes in water to membrane transfer free energy with experiment and other theoretical methods. Interestingly, our results suggest only a minor influence of lipid type on transfer energetics. Moreover, we achieve near-quantitative agreement with dimerization propensities for most mutations, with a few notable outliers. These simulations also allow estimation of the energetic contributions of methyl groups and hydrogen bonding to interface stabilization. Finally, we explore mutation-induced changes in helix-helix geometry, offering structural insights into how side-chain chemistry and lipid context modulate membrane protein association.

## Materials and methods

### Thermodynamic cycle

**Figure 1:**
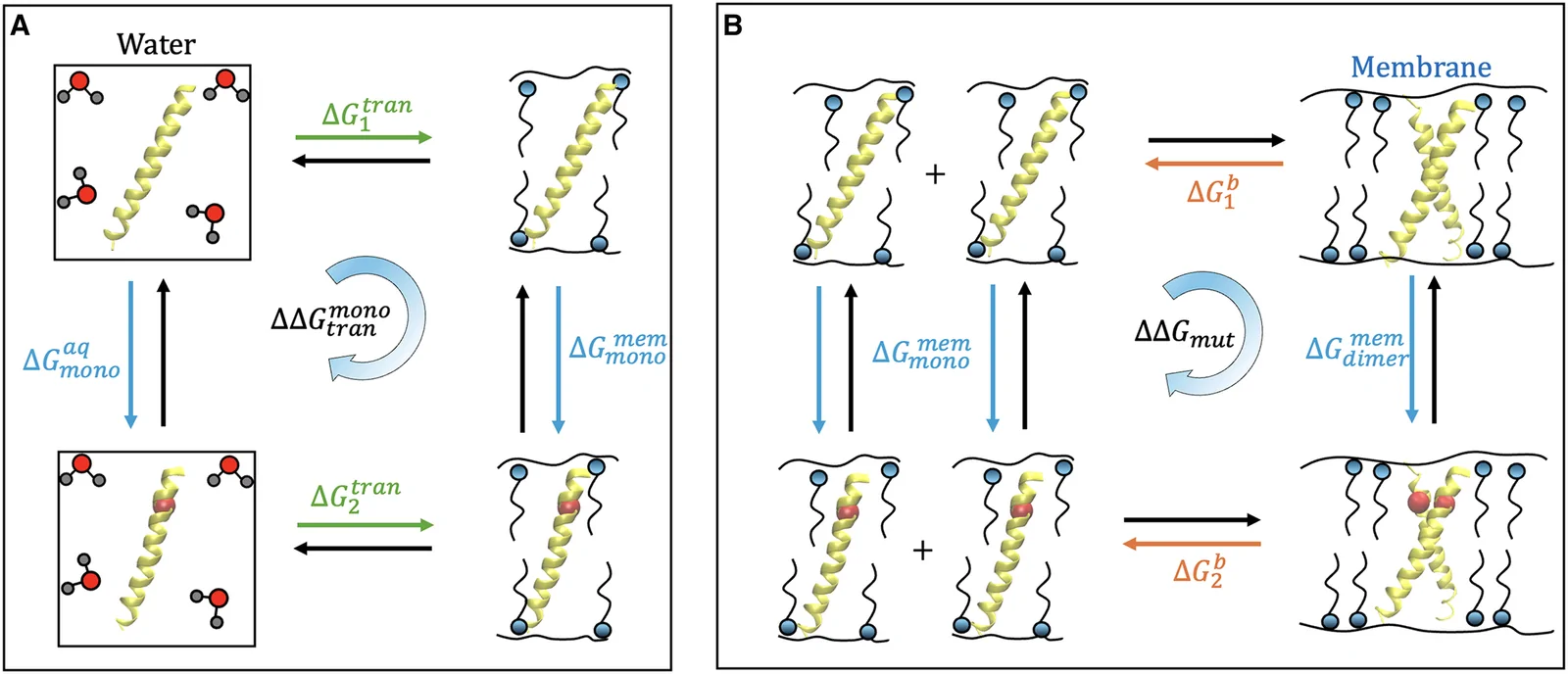
Thermodynamic cycles used to simulate the free energy change associated with (*A*) monomer transfer from aqueous solution into the membrane (Stage 1) and (*B*) dimerization of transmembrane (TM) GpA (Stage 2). Alchemical single-point mutations of TM GpA were performed for monomer and dimer in water and membrane, yielding the free energy changes $\Delta G_{mono}^{aq}$, $\Delta G_{mono}^{mem}$, and $\Delta G_{dimer}^{mem}$. Based on the zero-sum condition of the thermodynamic cycle Eq. (1), the transfer and dimerization free energy change $\Delta \Delta G_{tran}^{mono}$ and ΔΔ*G*_*mut*_ upon mutations were calculated. Mutation sites are highlighted as red spheres.

Thermodynamic cycles are constructed (Fig. 1) with three assumptions: 1) the dimer and monomer correspond to distinct stable states; 2) the peptides remain in helical conformations in the dissociated monomeric form, and 3) a two-stage model with monomer-insertion and dimerization representing separate independent processes (25). Using the “zero-sum” property of the cycle, the partitioning probability of helical mutated monomer (stage 1) $\Delta \Delta G_{tran}^{mono}$ and the dimerization propensity of mutated dimer relative to the wild-type (wt) can be predicted by the relative transfer free energy $\Delta \Delta G_{tran}^{mono}$ and the relative binding free energy upon mutation ΔΔ*G*_*mut*_:

(1) $$\Delta \Delta G_{tran}^{mono}=\Delta G_{2}^{tran}-\Delta G_{1}^{tran}=\Delta G_{mono}^{mem}-\Delta G_{mono}^{aq}$$

(2) $$\Delta \Delta G_{mut}=\Delta G_{2}^{b}-\Delta G_{1}^{b}=\Delta G_{dimer}^{mem}-2\Delta G_{mono}^{mem}\text{,}$$

where the alchemical transformation is simulated on the peptide in water and membrane to calculate $\Delta G_{mono}^{aq}$, $\Delta G_{mono}^{mem}$, and $\Delta G_{dimer}^{mem}$.

For a comparison with experimental data, it is important that the experimental measurement takes possible differences in the concentration of the monomers due to the mutation into account (e.g., TOXCAT assay (21)). In the TOXCAT essay, this is included by the Western plot band intensities, presenting the concentration of wt (*W*_*wt*_) or mutant (*WB*_*mut*_) fusion monomer proteins in the membrane. The enzymatic *CAT* activity measured in experiments for the mutant (*CAT*_*mut*_) or wt (*CAT*_*wt*_) constructs is normalized with respect to the cells expressing the constructs. Considering the experimental apparent changes in free energy from stage 2, $\Delta \Delta G_{mut}^{TOXCAT}=-RT\;\ln\left(\frac{CAT_{mut}}{CAT_{wt}}\right)+2RT\;\ln\left(\frac{WB_{mut}}{WB_{wt}}\right)$. The alchemical calculations of $\Delta G_{dimer}^{mem}$ and $\Delta G_{mono}^{mem}$ from Eqs. (1) and (2) are equivalent to the measurement of CAT activity (Eq. (3)) and WB (Eq. (4)), respectively, by the following:

(3) $$\Delta G_{dimer}^{mem}=-RT\;\ln\left(\frac{CAT_{mut}}{CAT_{wt}}\right)$$

(4) $$\Delta G_{monomer}^{mem}=-RT\;\ln\left(\frac{WB_{mut}}{WB_{wt}}\right)$$

### System construction

The simulated GpA-TMD dimer structure in form of right-handed parallel *α*-helix is provided by x-ray crystallography in lipidic cubic phase (36) (PDB: 5EH4). The monomer is derived from the dimer structure, giving the sequence (37) EPEITLIIFGVIAGVIGTILLISYGIRRLC. Using CHARMM-GUI (38), the wt peptides were embedded into the monolipid bilayer of chosen types POPC (16:0/18:1), POPE (16:0/18:1), PLPC (16:0/18:2), and PYPC (16:0/16:1) with 90 lipids per leaflet. The system is neutralized in a 0.15 M NaCl bath with protonation state choice of amino acids reflecting a pH of 7.0. The engineered residue of the GpA crystal structure is assigned to cysteine to maintain the reference to the fluorescence study (36). The periodic simulation box has a natural semi-isotropic volume fluctuation of biological membranes (39) and a dimension of ∼75 × 75 × 90 Å^3^ with ∼56,339 atoms. The z-dimension of the lipid bilayer is ∼40 Å. For the simulation of the GpA monomer in water, similar physiological conditions to the membrane are set up with a square simulation box of ∼70 × 70 × 70 Å^3^.

### Alchemical free energy calculation

The dual-topology alchemical transformations (40) were initiated from 100-ns-equilibrated wt GpA trajectories in a membrane environment. During these simulations the root mean-square deviation (RMSD), dimer crossing angle, area per lipid, and membrane thickness reached mean plateau levels (Fig. S1). Mutations were introduced in silico into both monomeric and dimeric states, with substitutions in the dimer applied symmetrically to each helix. For glycine mutations, the backbone *C*_*α*_ atom was treated as “annihilated” or “exnihilated” rather than “staying” in order to preserve appropriate bonded parameters given glycine’s unique dihedral potential. Each system was energy-minimized for 5000 steps and equilibrated for 2 ns before production. Relative free energy changes were estimated via free energy perturbation (FEP) (40,41). Peptides were simulated unrestrained in the membrane due to their stable helical structure, whereas monomers in water were restrained by a weak 0.25 kcal/mol/Å^2^ harmonic potential on the three terminal *C*_*α*_ atoms to preserve helicity and prevent collapse driven by hydrophobic exposure during the alchemical transformation.

Alchemical switching employed a soft-core potential scheme and separate scaling (42), with electrostatic and van der Waals interactions decoupled using alchElecLambdaStart = 0.5 and alchVdwLambdaEnd = 1.0, respectively (43). To suppress steric clashes at the endpoints, a van der Waals shift coefficient of alchVdwShiftCoeff = 5.0 was applied (42). Parameter sensitivity analyses on two representative mutations of small and larger perturbation (V80A and I76S; Fig. S2) showed negligible influence on calculated free energies, although sampling efficiency varied depending on the potential switching schedule.

All transformations were carried out over 20 equally spaced *λ*-windows (Δ*λ* = 0.05), each preceded by 0.5 ns of equilibration. Both forward and backward directions were sampled and analyzed using the multistate Bennett acceptance ratio as implemented in ParseFEP (31,43). Convergence was assessed by progressively extending the sampling time per window (*t*_*λ*_) from 1 ns up to 20 ns, depending on mutation. Monomers required less sampling than dimers, as expected from a smaller conformational phase space. Final free energies are reported as averages of the last three independently converged simulations, with *t*_*λ*_ inclusive of 0.5 ns equilibration time per *λ*-window.

### MD simulation

All simulations were performed in NAMD 3.0b6 (43) with the CHARMM36m force field (44) and the explicit TIP3P water model (45). The built systems were minimized using conjugate gradient descent methods with 5000 steps and then equilibrated in *NPT* ensemble under a constant temperature of 310 K and a pressure of 1 bar, controlled by the Langevin thermostat (46) and Langevin semiisotropic piston (47) with a friction coefficient of 1 ps^−1^. The trajectory is updated after each time step of 2 fs using the Verlet r-RESPA algorithm (48). The trajectory frames were recorded every 0.1 ns for simulations shorter than 300 ns. For simulations longer than 300 ns, a frequency of 0.5 ns was chosen.

The nonbonded interactions are controlled by smoothing the LJ potential over the distance range of 10 Å to 12 Å using force-based switching function (43), whereas the PME method with a grid spacing of 1.2 Å was employed to involve the long-range electrostatic interactions (38). The hydrogen bond length between heavy and hydrogen atoms is constrained by the SHAKE algorithm, except for water, for which the SETTLE algorithm was applied (49). The trajectory analysis is performed mainly by MDAnalysis (50), VMD tools (51), and other available numerical Python libraries. The molecular pictures are produced by VMD (51). The average properties were calculated from the last 50 ns of the trajectories. To treat the splitting artifact of the dimer due to the periodic boundary condition, we use the reversible unwrapping algorithm (52).

## Results and Discussion

**Figure 2:**
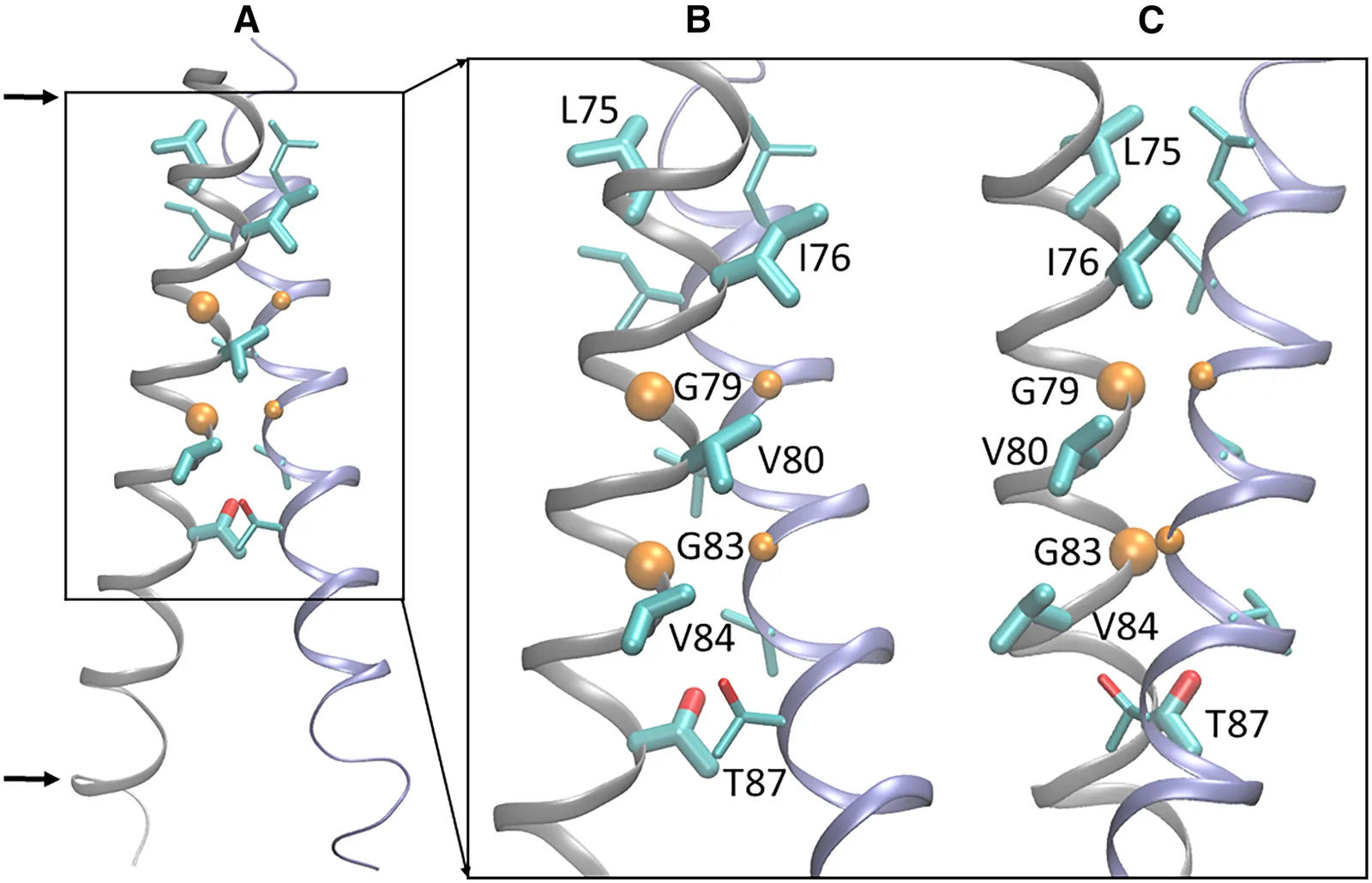
Structural details of GpA dimer. (*A*) Parallel right-handed GpA dimer consisting of two identical 30-residue monomers with the sequence (37) EPEITLIIFGVIAGVIGTILLISYGIRRLC. The PDB code is 5EH4 (36). The approximate boundaries between the lipid bilayer and the aqueous phase are indicated by arrows. (*B*) Stick representation of important dimer interface residues, including labels. Bold sticks are for chain A and fine sticks for chain B. (*C*) Same as (*B*) but with a view rotated by 90° around the membrane normal.

Mutations within the membrane-spanning helix of GpA (Fig. 2) can alter both the free energy of transfer from an aqueous environment into the bilayer and the stability of GpA dimerization. Here, we investigated the impact of single-point mutations on these two processes and examined how different membrane lipid compositions modulate their effects by means of relative free energy change. Dimerization free energies were determined primarily in POPC and POPE bilayers, representative of the dominant lipid species in eukaryotic and prokaryotic membranes, respectively. To characterize the dimer, its crossing and tilt angles were calculated as described by Petrache et al. (18). The buried surface area (BSA) was calculated by subtracting twice the solvent-accessible surface area (SASA) of the monomer from the SASA of the dimer, providing a quantitative measure of the packing at the dimer interface.

### Stage 1: Change in relative partitioning probability for *α*-helix mutations from water to membrane

In nature, membrane proteins are typically expressed and inserted directly into the membrane. However, in mutagenesis studies, often the insertion needs to be considered due to extracellular protein expression. To quantify the impact of single-residue mutations on membrane insertion energetics, we employed alchemical free energy calculations to compute the relative transfer free energies $\left(\Delta \Delta G_{mono}^{trans}\right)$ of a GpA monomer from water into various lipid environments such as POPE, POPC, PLPC, and PYPC (Fig. 1 *A*). Among these, PLPC has increased membrane fluidity due to its polyunsaturated acyl chains, whereas PYPC forms a thinner bilayer due to its shorter chains (53). In all systems, the free energy estimates converged within 200 ns.

**Figure 3:**
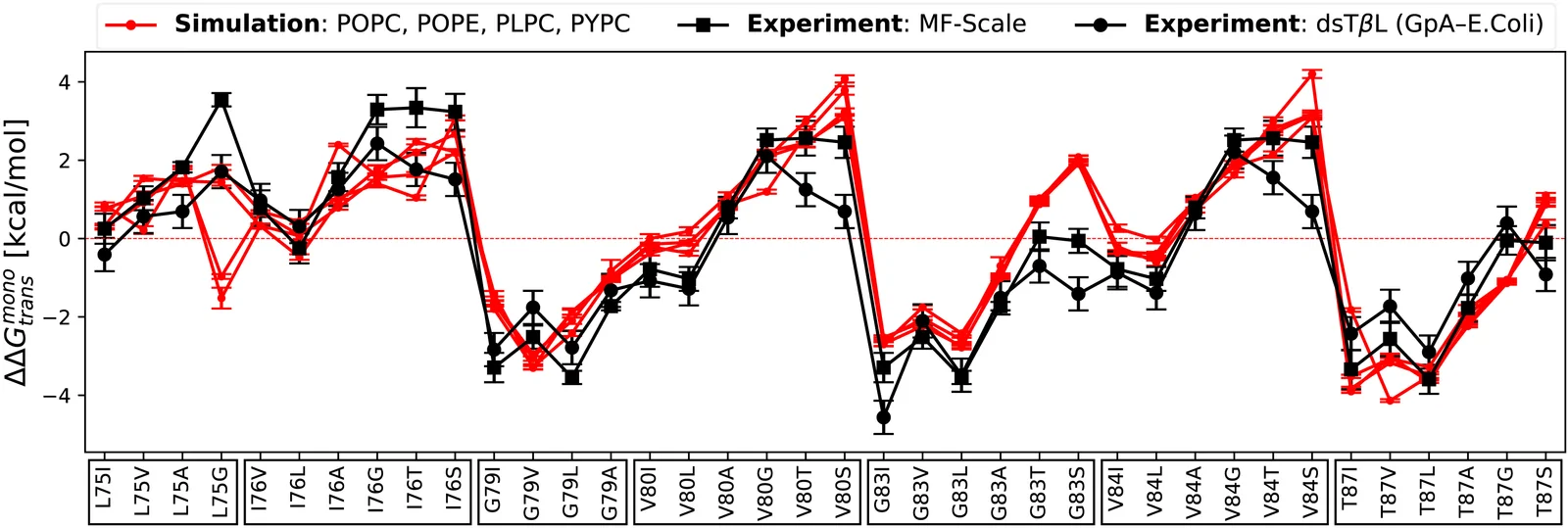
Relative transfer free energy of the GpA monomer $\left(\Delta \Delta G_{trans}^{mono}\right)$ simulated in various homogeneous lipid bilayers: POPC (16:0/18:1), POPE (16:0/18:1), PYPC (16:0/16:1), and PLPC (16:0/18:2). Simulation results are compared with experimental data from dsT *β* L assays (6) and the MF hydrophobicity scale (54). Mutations are ordered according to the reduced Kyte-Doolittle hydrophobicity scale of the target residue (56). Error bars represent errors of the mean.

As shown in Fig. 3, our calculated transfer free energies correlate well with experimental partitioning scales, both the empirical MF-scale and the dsT *β* L measurements in bacterial membranes, with high statistical significance (see also Fig. S3). The MF-scale experiment was generalized for diverse membrane protein architectures (54), while dsT *β* L assays improved the insertion detection by considering bacteria survival-insertion relationship through a *β*-lactamase readout on both *β*-barrel and GpA-helix (6). Both experiments measure the relative free energy change of insertion of individual amino acid side chains in vivo and thus are comparable to our simulation. The agreement is particularly strong for apolar-to-apolar mutations. To note, for mutations such as L75G in PLPC and PYPC, we observed disruption of the helical structure near the membrane boundary, likely due to the loss of hydrophobic anchoring from the leucine side chain (4,55), exacerbated by the higher dynamics at the water-bilayer interface in these lipids compared with POPE or POPC. These effects raise a sampling problem in the free energy of the L75G in these more dynamic environments (Fig. S3 inset).

For apolar-to-polar mutations (e.g., Ser and Thr substitutions), deviations from experimental values reached up to ∼1–2 kcal/mol, especially for mutations near the membrane center. However, these discrepancies are smaller than those predicted by the GeTFEP model based on weighted potentials (57) and are within its error margins (Fig. S4). Interestingly, our calculated free energies show smaller deviations from the MF-scale than the dsT *β* L experimental results, highlighting that biological membranes may facilitate membrane insertion via intermediate mechanisms (e.g., interface anchoring by polar residues, cotranslational insertion, or translocon assistance) that are not captured by the two-stage model assumed in our simulations (Fig. 2) (4,55,58).

Another important observation is that the transfer free energies for mutations located at the membrane center are relatively insensitive to lipid type, even for apolar-to-polar substitutions indicating an important role of the hydrophobic core for the thermodynamic cost of insertion (56,59). However, for mutations near the water-membrane interface (e.g., L75, I76, T87), lipid-dependent effects are more pronounced, with variations up to ∼1 kcal/mol. This is consistent with the hypothesis that lipid headgroup properties influence residue energetics near the interface (55).

Specifically, the POPC lipid environment reduced the transfer free energy of the I76T mutant relative to POPE (Fig. S4), suggesting a more favorable interaction between the Thr side chain and the larger PC headgroup. This effect was not observed for the I76S mutant, possibly due to weaker or less specific interactions of the smaller Ser side chain, pointing to a size-dependent modulation of residue-lipid interactions. Furthermore, the thinner bilayer of PYPC lowered the transfer free energy of both I76S and I76T mutants, in line with previous findings by Jakob et al. for a *β*-barrel protein in similarly thin DPPC membranes (60). Notably, $\Delta \Delta G_{mono}^{trans}$ values in PLPC were comparable to those in POPE and POPC for most mutations, suggesting that acyl chain saturation has a minor influence on the insertion thermodynamics of stable *α*-helices (60).

### Stage 2: Effect of mutations on the GpA dimerization stability in homogenous membranes

Exploiting the two-stage model (Fig. 1), we performed alchemical free energy simulations of the GpA dimer embedded in lipid bilayers. This approach yields the relative membrane dimerization free energy, ΔΔ*G*_*mut*_, quantifying the stability change upon point mutations. Given the experimentally determined absolute transfer free energy of wt GpA of ∼ −12 kcal/mol (61), the relative free energy of the monomer with single-point mutations does not exceed 3 kcal/mol and thus has some extent of successful insertion probability (see Fig. 3). Here, we choose POPC and POPE lipid representatives of mammalian and bacterial membranes to facilitate experimental validation and design. For nearly all systems, convergence was achieved within 500 ns.

In most cases, we observed excellent agreement between forward and backward alchemical transformations and good convergence of ensemble averages versus simulation time, as shown in example cases V80A of somewhat small transformation and I76S of somewhat larger transformation (Fig. S5). However, for mutations of the small Gly residues in the GxxxG motif to bulkier residues, we sometimes observed disruption of the dimer. Since we are interested in the change in free energy in the bound state, we applied in these cases a weak positional restraint (force constant of 0.1 kcal/mol/Å^2^ on four C_*α*_ atoms of terminal residues 96 and 77) to achieve convergence from the preserved native-like dimer geometry (example case G83T Fig. S7). For transformations G79I and G83I, even the weak restraint on terminal residues did not prevent conformational changes due to steric clashes. Hence, the backward simulation starts from a distorted helix that does not reach an undistorted dimer structure (for the back transformation to G). It leads to hysteresis between forward and backward results (see Fig. S6). Nevertheless, the result of the forward transformation may still provide a reasonable estimate because it starts from an intact dimer.

**Figure 4:**
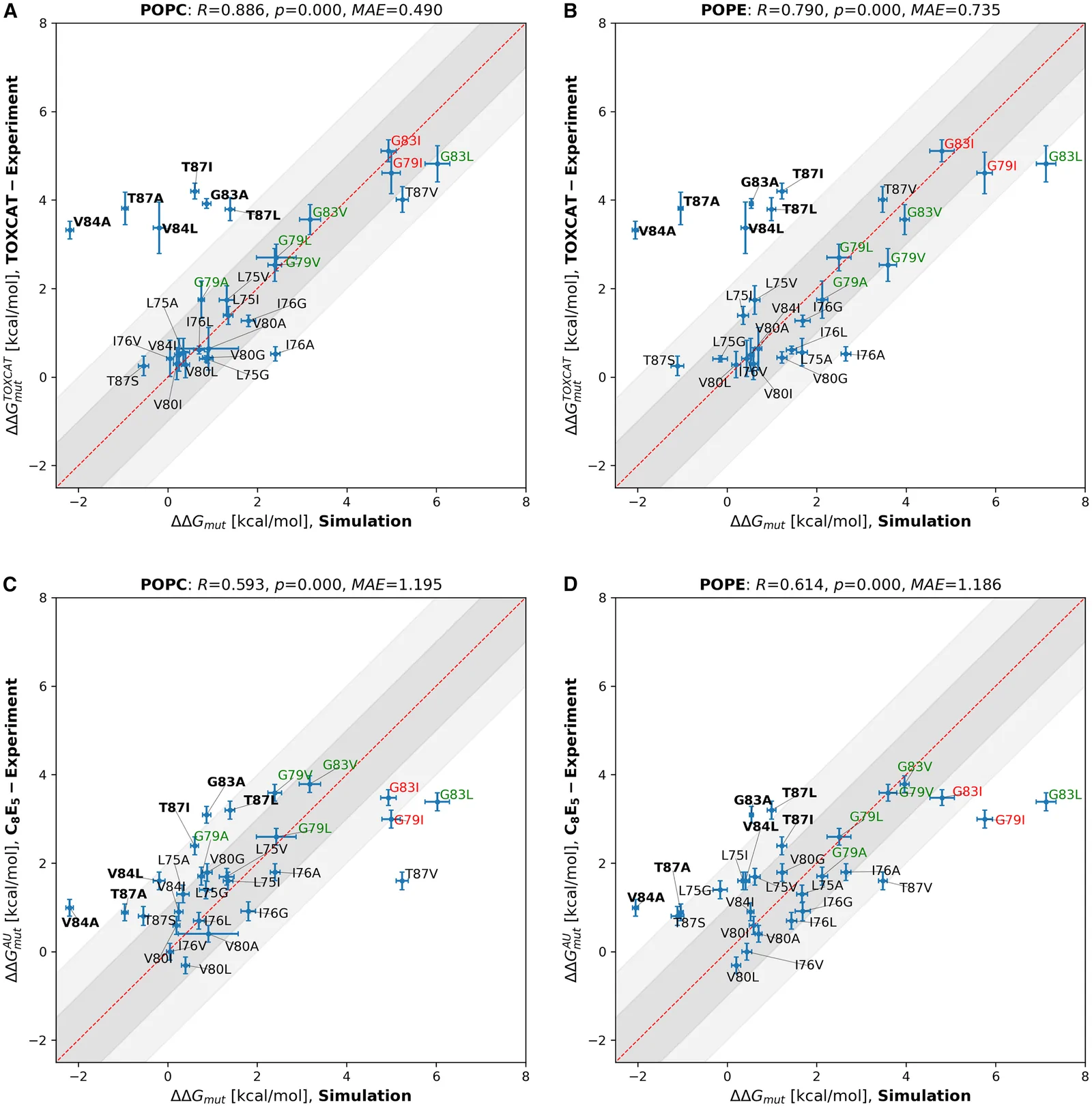
Correlation of relative dimerization free energy changes between experiments and simulation. Experimental data were obtained from TOXCAT assays in *E. coli* membrane (21) and sedimentation equilibrium analytical ultracentrifugation in C_8_E_5_ micelles (15,19,62,63,64)). Simulated values are shown for dimerization in (*A, C*) POPC and (*B, D*) POPE bilayers. In (*A*) and (*B*), the Pearson correlation coefficient (*R*), statistical significance (*p*), and mean absolute error (*MAE*) were computed excluding six outlier mutations (shown in bold). (*C*) and (*D*) include all 28 available mutations. Green-highlighted data points correspond to mutants with large conformational changes that were positionally restrained in the bound state using a weak force constant of 0.1 kcal/mol/Å^2^ on four C_*α*_ atoms (residues 96 and 77). Red-highlighted mutants dissociated apparently during the transformation and did not converge; their free energies were estimated from a single forward transformation only. Shaded regions indicate a free energy difference of 1–2 kcal/mol.

As shown in Fig. 4, our calculated ΔΔ*G*_*mut*_ correlates well with the apparent experimental free energy changes $\Delta \Delta G_{mut}^{TOXCAT}$ for both membrane POPC and POPE with visible outliers V84A, V84L, T87A, T87L, T87I, and G83A. Excluding these outliers, the correlation of the remaining 22 mutations becomes obvious by an improvement from *R* = 0.243, *p* = 0.006, mean absolute error (*MAE)* = 1.202 kcal/mol to *R* = 0.886, *p* = 0.000, *MAE* = 0.49 kcal/mol for POPC bilayer and from *R* = 0.156, *p* = 0.008, *MAE* = 0.668 kcal/mol to *R* = 0.790, *p* = 0.000, *MAE* = 0.735 kcal/mol for POPE one. Moreover, the regression analysis (Fig. S8) shows a scaling below 20% and an energy biasing below 0.2 kcal/mol of experimental data by simulation data, resulting in good correlation overall. Comparing to the entire experimental data of the same quantity ΔΔ*G*_*mut*_ (Fig. S9) measured in micelle environments (Fig. 4 *C* and *D*), the outliers appear to be partially eliminated, again emphasizing the dependency of dimer configuration of these mutants on the environment and experimental methodology (21). The source of discrepancies could be inferred from the assumptions, detection limit of chimeric construct, nonideal membrane mimetics, etc. (15,19,62,63,64).

Considering only the mutations with exceptional correlation to TOXCAT experiment, the MAE is 0.444 kcal/mol and 0.668 kcal/mol for POPC and POPE membrane, respectively, which is in the common range of force field accuracy and in the successful range of alchemical free energy calculation in drug design (65,66,67). During the alchemical transformation, the *C*_*α*_-RMSD of all converged mutants in the POPC membrane remains stable for both forward and backward directions, as shown in Figs. S10 and S11 (POPE membrane is similar and not shown), ensuring the assumption of the thermodynamic cycle (Fig. 1). For the mutated dimer and monomer, the RMSD does not exceed 3 Å and 2 Å, respectively. These values are in line with the common RMSD of wt-GpA from the structure derived from NMR experiments (19,26,36,68).

### Energetic contribution of hydrogen bond and methyl group in dimer stabilization

**Figure 5:**
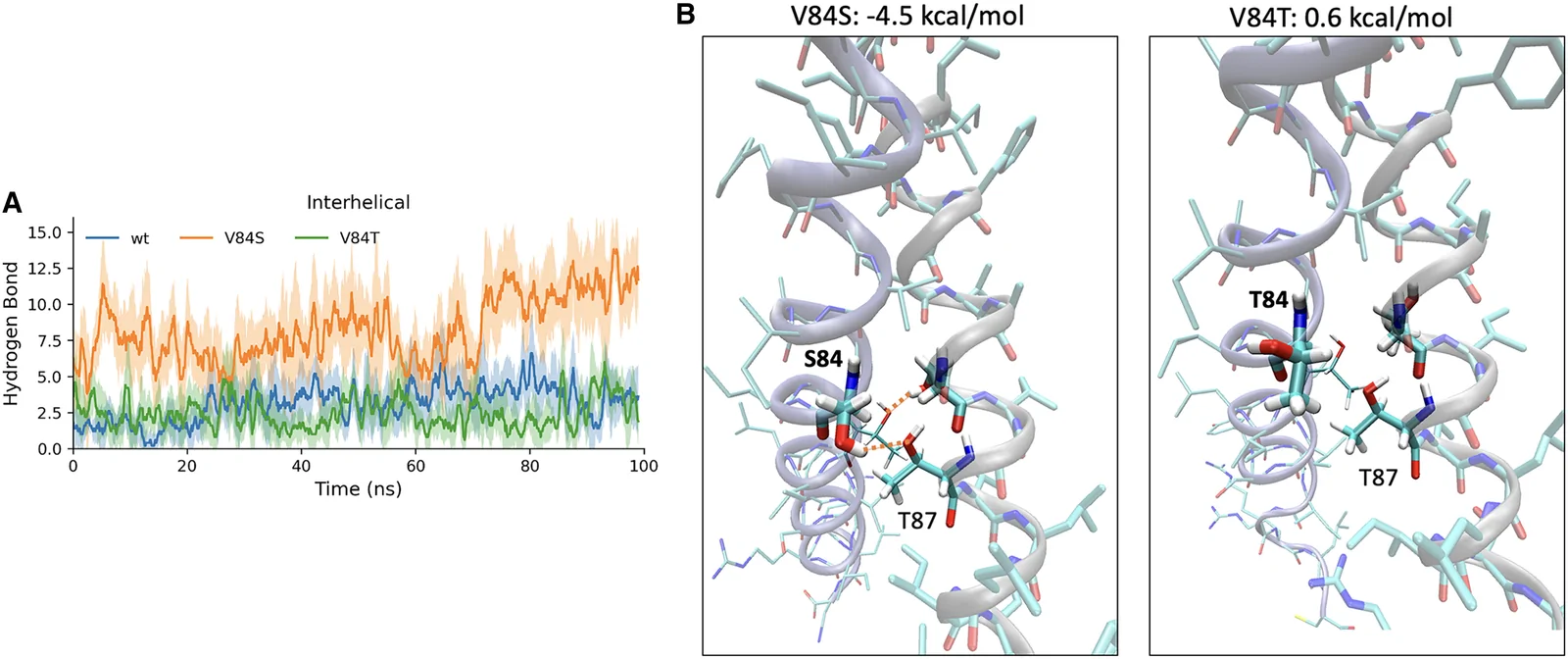
Dimer stabilization through enhanced packing mediated by hydrogen bond formation. (*A*) Dynamics of interhelical hydrogen bond count of GpA-wild-type (wt) and the V84S and V84T mutants (see also Fig. S12). The shaded regions represent the standard deviation. (*B*) In the V84S mutant, a stable hydrogen bond (*orange dashed line*) forms between the hydroxyl group of Ser84 (fat chain) and the hydroxyl group of Thr87 (thin chain), which is absent in the V84T mutant. The structural snapshots correspond to the final frames of a 100-ns equilibration simulation.

C_*α*_–H ··· O interactions between interhelical residues, such as T87 and V84 in GpA, have been proposed to contribute to dimer stabilization (13,20,69). Although T87 mutants have been widely studied, the contribution of V84, particularly with polar substitutions, has received less attention. Here, we find that the V84S mutation significantly stabilizes the dimer by approximately −4 kcal/mol, owing to the formation of strong, symmetric and stable hydrogen bonds between S84 and T87 across the two monomers (increased hydrogen bond count illustrated in Figs. 5 *A* and S12 *A*, binding geometry indicated for a snapshot in Fig. 5 *B*). In contrast, the V84T mutation does not confer similar stabilization, due to steric hindrance that prevents optimal hydrogen bond geometry, despite the presence of an –OH group (Fig. 5 *B*). This underscores that hydrogen bonds contribute to dimer stabilization only when sterically accommodated by the packing environment, supporting the hypothesis that van der Waals interactions remain the dominant stabilizing force (14,15,16).

Structurally, the V84S mutant adopts a different conformation than V84T with a larger crossing angle (Fig. S12 *B* and *C*). The hydrogen bond-structure association also explains why mutations at V80 (e.g., ΔΔ*G*_*mut*_(V80S) = 1.26 kcal/mol, ΔΔ*G*_*mut*_(V80T) = 0.61 kcal/mol in POPC) are destabilizing: no compensating hydrogen bonds can form to replace the native G83-V80 backbone interaction (13,20,69). Taken together, these results support the hypothesis that hydrogen bonding can enhance dimer stability but only within the structural constraints of interhelical packing and membrane coupling (10,20,35). Finally, the tolerance of V84 and V80 to both small and large side-chain substitutions supports the existence of broader motif variants such as G[Lg]xxG[Lg]xx[Sm] or G[Sm]xxG[Sm]xxT in natural dimerization interfaces (14).

In comparison to V84S, the V84A mutant yields a moderate stabilization of approximately −2 kcal/mol, lacking the hydrogen bond but retaining favorable packing. From this, we infer that the energetic contribution of a single interhelical hydrogen bond is approximately 1 kcal/mol, consistent with the difference between V84A and V84S, which differ only by the presence of a hydroxyl group.

We also assessed the energetic impact of methyl group removal (Table S1; Fig. S9). In the same POPC lipid, mutations L75V (ΔΔ*G*_*mut*_ = 1.3 kcal/mol) and V80A (ΔΔ*G*_*mut*_ = 0.64 kcal/mol) each lead to a destabilization of approximately 0.5 kcal/mol, whereas I76V (ΔΔ*G*_*mut*_ = −0.3 kcal/mol) and T87S (ΔΔ*G*_*mut*_ = −0.5 kcal/mol) are mildly stabilizing. Moreover, the interplay between lipid environment and methyl group removal is observed in POPE, where I76V (ΔΔ*G*_*mut*_ = 0.4 kcal/mol) is slightly destabilizing, whereas T87S (ΔΔ*G*_*mut*_ = −1.4 kcal/mol) is more stabilizing. Taken together, these results indicate that individual methyl groups contribute modestly to either dimer stabilization or destabilization, depending on both the mutational position and the lipid environment.

### Context-dependent effects of GxxxG mutations on transmembrane dimer stability

The sequence specificity of the GxxxG motif in driving transmembrane helix dimerization has been debated across several studies (14,15,16). We therefore investigated the mutational sensitivity of this motif using alchemical free energy simulations. In agreement with experimental findings, substitution of glycine residues with larger side chains destabilizes the dimer by 2.5–4 kcal/mol (Fig. 4; Table 1). However, alanine substitutions at positions G79 and G83 retain partial dimer stability, with ΔΔ*G*_*mut*_< 2.5 kcal/mol, indicating that small side-chain replacements are tolerated to some degree.

**Table 1:** Free energy changes ΔΔ*G*_*mut*_ (kcal/mol) (Fig. 1) of mutated GpA-dimer relative to wildtype in different membranes

	Simulation				Experiment	
	POPC	POPE	PLPC	PYPC	TOXCAT (21)	GALLEX (12)
G83I	4.93 ± 0.17	4.80 ± 0.27	1.58 ± 0.18	2.19 ± 0.11	5.11 ± 0.53	2.15
G83T	2.91 ± 0.23	1.23 ± 0.12	1.29 ± 0.10	0.65 ± 0.09		
G83A	0.87 ± 0.09	0.54 ± 0.04	−1.03 ± 0.31	−1.34 ± 0.05	3.92 ± 0.22	1
G79A	0.75 ± 0.07	2.12 ± 0.12	0.28 ± 0.40	0.55 ± 0.06	1.75 ± 0.68	1.2

The mutations were made at the GxxxG motif. The experimental values are given for comparison.

**Figure 6:**
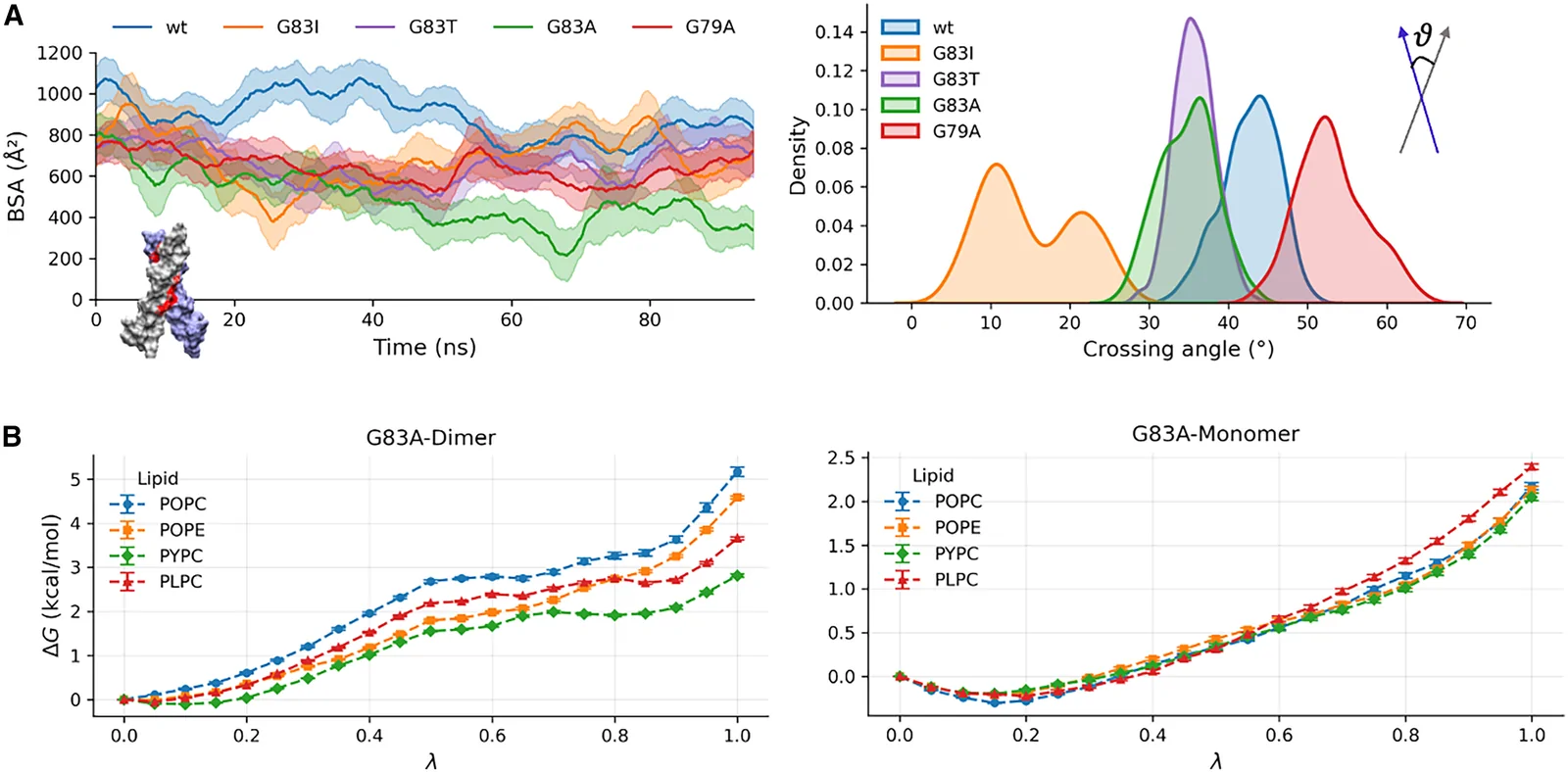
Analysis of GxxxG motif mutations. (*A*) Dynamics of buried surface area (BSA, *illustrated red interface surface between the monomer in the inset*) and crossing angle density distribution of GpA dimer mutants (*see θ in the inset*) compared with the wild-type (wt) in POPC bilayer. (*B*) Alchemical free energy change in the forward transformation of G83A dimer and monomer mutant in different lipid bilayers. Error bars represent errors of the mean.

In the same POPC bilayer, despite comparable destabilization energies, G79A and G83A mutants exhibit distinct structural responses, as indicated by different lipid exposure levels and overall dimer geometries (Fig. 6 *A*). Locally, positional sensitivity is reflected in hydrogen-bonding patterns: G79A retains stabilizing interhelical hydrogen bonds (G83–A79, T87–T87), whereas G83A loses native contacts (Fig. S13), leading to a local rearrangement of neighboring residues (Fig. S14). Regarding lipid interactions, the G83A mutant forms more hydrogen bonds with lipid headgroups, resulting in greater tilt-angle flexibility (Fig. S15). This may also explain why G83A is the only GxxxG mutant that converges in free energy calculations without positional restraints. In POPE bilayers, G79A disrupts packing more than G83A, by approximately 1.5 kcal/mol, whereas this is not observed in POPC, suggesting a specific role of the PE headgroup due to hydrogen bonding with charged terminal residues. Taken together, G83A appears to favor lipid-protein interactions as the main contributor to dimer stabilization over G79A, although this effect is position dependent.

Observations of small residue mutation support the idea that lipid-protein coupling can mitigate the entropic cost of disrupting close packing at the GxxxG motif (6,16). To probe this further, we simulated some representative GxxxG mutations in PLPC and PYPC membranes (Table 1). Surprisingly, G83A showed mild stabilization (∼−1 kcal/mol) in PLPC/PYPC, whereas G79A did not. Moreover, both PLPC and PYPC stabilized the dimer in G83I and G83T mutants, otherwise disruptive in POPC/POPE, suggesting that increased membrane fluidity can buffer the packing and entropic perturbations introduced by larger residues. This supports a role for membrane-mediated entropy in dimer stabilization (14,22,29,68). In addition, the effect of lipid on G83A dimer stability is indirect due to different dimer conformation, since no significant effect on the monomer is observed (Fig. 6 *B*).

Comparison of G83I and G83T highlights the role of hydrogen bonding: while Ile disrupts packing significantly, Thr partially compensates via interhelical and terminal Glu–Glu hydrogen bonds (Fig. S13), preserving dimer geometry (Fig. 6 *A*). However, both mutants show similar lipid exposure (Fig. 6 *A*), and G83I cannot form hydrogen bonds, underscoring that side-chain polarity and H-bonding are crucial for accommodating disruptive mutations, as also seen in the case of V84S. Taken together, the results align with prior findings that van der Waals interactions dominate GxxxG-mediated dimerization but can be modulated by hydrogen bonds and lipid coupling (14,15,35).

Overall, our results demonstrate that the structural and energetic consequences of mutating the GxxxG motif are highly position specific, side chain dependent, and membrane sensitive. Although some mutations (e.g., G83A) benefit from entropic and lipid-mediated compensation, others (e.g., G83I) disrupt local packing too severely for such compensation to be effective. These findings reconcile conflicting experimental observations between detergents and lipid bilayers: for instance, G83A in POPC bilayers shows smaller crossing angles configurations favored in detergents (27). This suggests that bilayer flexibility allows structural adaptation upon mutation, unlike the more rigid micelle environments, which restrict conformational relaxation and exaggerate destabilization effects (33,61).

Thus, although (small)xxx(small) motifs promote dimerization via van der Waals contacts, their tolerance to mutation depends critically on local packing geometry, hydrogen bond rearrangement, and lipid-protein coupling. This context sensitivity explains why similar mutations can result in different outcomes at G79 and G83 and why biological membrane complexity must be considered when interpreting mutational effects (4,22,70).

## Conclusion

Over the past decades, thermodynamic stability of membrane protein dimerization has been experimentally performed under diverse conditions and methodologies, making it difficult to extract generalizable principles (37). In this work, we present a computational framework to evaluate the thermodynamic stability of the GpA dimer using atomistic alchemical simulations, an approach known for its rigor but not previously applied to membrane-embedded protein complexes. The accuracy of our calculations is enhanced through the multistate Bennett acceptance ratio, and the results show strong agreement with experimental data, achieving high statistical significance. Notably, the calculated free energy changes converge within submicrosecond timescales (<1*μ* s), outperforming traditional PMF methods in both efficiency and resolution. This efficiency stems from the fact that the unfolded state in membrane proteins is typically a well-defined helical conformation, which avoids the complexity of highly dynamic unfolded ensembles seen in soluble proteins. Furthermore, our simulations directly reveal substantial conformational changes and increased dissociation propensity in the GpA dimer upon mutation of the GxxxG motif, findings consistent with experimental sensitivity to these positions.

Using the protocol, we estimate the energetic contributions of hydrogen bonding and methyl group interactions to dimer stabilization to be approximately 1.0 kcal/mol and 0.5 kcal/mol, respectively, using Ser and Thr substitutions at key interfacial residues. These results reinforce the notion that van der Waals interactions, rather than hydrogen bonds alone, are the primary driving force of dimer association, with hydrogen bonds contributing only under favorable packing constraints, as exemplified by V84S and V84T. Additionally, we identify an indirect stabilizing effect of lipid fluidity on dimerization, particularly in mutants such as G79A and G83A. Lipids with higher lateral mobility (e.g., PLPC, PYPC) contribute up to ∼3 kcal/mol in stabilization by compensating for entropic penalties, emphasizing the environment-sensitive nature of the GxxxG motif’s role in dimerization. For example, it is known that cholesterol in a membrane influences GpA association, and future free energy simulation studies could be useful to elucidate the molecular details of how cholesterol mediates GpA dimerization.

## Data and code availability

The MD and free energy simulation input files, representative output data, and analysis scripts supporting the findings of this study have been deposited in Zenodo and are publicly available under the accession number https://doi.org/10.5281/zenodo.18089109 as of the article publication date. Due to their large size, full raw simulation trajectories are not included in the public repository but will be shared by the corresponding author upon reasonable request.

## Acknowledgments

The authors thank N. Halbwedl, C. Sustay, and Dr. P. Quoika for useful discussions. This work was financially supported by the 10.13039/501100001659Deutsche Forschungsgemeinschaft (DFG Za153/29-1) and by a local compute cluster (partially funded by 10.13039/501100001659DFG INST 95/1610-1 FUGG). We acknowledge additional HPC resources provided by the Erlangen National High Performance Center (NHR@FAU) of the Friedrich-Alexander-Universität Erlangen-Nürnberg (FAU) under the NHR project b118bb.

## Author contributions

C.V.Q. performed simulations, wrote programs and scripts, performed visualization, analyzed data, and wrote the paper draft. M.K. designed the project, supervised computational work, wrote the paper draft, and wrote the final manuscript version. M.Z. provided resources, obtained funding, designed the project, supervised work, and wrote the final manuscript version. C.V.Q and M.K. and M.Z. analyzed results and reviewed the manuscript.

## Declaration of interests

The authors declare no competing interests.

## Supporting material

Document S1. Figures S1–S15 and Tables S1 and S2

Document S2. Article plus supporting material
