# Supplementary material for: Membrane insertion and dimerization of glycophorin-A mutations studied by free energy simulations
Source: Biophys J. 2026 Jan 5;125(3):950–61. doi: 10.1016/j.bpj.2025.12.039 (PMC13351972; doi:10.1016/j.bpj.2025.12.039)
Supplement: Document S1. Figures S1–S15 and Tables S1 and S2 [file mmc1.pdf]

Biophysical Journal, Volume *125*

Supplemental information

Membrane insertion and dimerization of glycophorin-A mutations studied by free energy simulations

Cong Van Quy, Martin Kulke, and Martin Zacharias

Supporting Information:
Membrane insertion and dimerisation of glycophorin-A mutations studied by free energy simulations

Cong Van Quy[1,2], Martin Kulke[1], and Martin Zacharias[1,*]

[1]Physics Department and Center of Protein Assemblies, Technical University of Munich, Garching, Germany
[2]Computational Biomedicine, Forschungszentrum Jülich, Germany
[*]Correspondence: zacharias@tum.de
[1]Physics Department and Center of Protein Assemblies, Technical University of Munich, Garching, Germany
[2]Computational Biomedicine, Forschungszentrum Jülich, Germany

A FREE ENERGY DATA AND CONVERGENCE ANALYSIS

Table S1: Alchemical free energy change of GpA dimer (ΔG^{mem}_{dimer}) and monomer ($\Delta G^{mem}_{monomer}$) in different bilayers, given in kcal/mol. Red color denotes dimer mutants that do not have intrinsic converged values, which, however, can be estimated by the accumulated forward free energy under consideration of the conformational change.

Mutation	POPC		POPE		PLPC	PYPC
	Dimer	Monomer	Dimer	Monomer	Dimer	Dimer
L75I	10.10 ± 0.07	4.38 ± 0.06	8.22 ± 0.09	3.93 ± 0.06		
L75V	10.66 ± 0.06	4.67 ± 0.11	10.84 ± 0.07	5.11 ± 0.07		
L75A	2.32 ± 0.11	0.99 ± 0.07	3.78 ± 0.08	1.05 ± 0.07		
L75G	-2.41 ± 0.12	-1.64 ± 0.06	-4.19 ± 0.11	-2.02 ± 0.09		
I76V	1.10 ± 0.06	0.70 ± 0.04	1.10 ± 0.06	0.33 ± 0.07		
I76L	-6.41 ± 0.09	-3.56 ± 0.06	-7.81 ± 0.07	-4.63 ± 0.07		
I76A	-2.99 ± 0.07	-2.70 ± 0.05	-2.92 ± 0.05	-2.79 ± 0.07	-1.76 ± 0.10	-1.49 ± 0.08
I76G	-5.87 ± 0.09	-3.84 ± 0.10	-5.25 ± 0.08	-3.47 ± 0.11		
I76S	-10.92 ± 0.09	-7.19 ± 0.06	-13.48 ± 0.11	-7.60 ± 0.07		
I76T	-11.97 ± 0.07	-6.79 ± 0.06	-8.21 ± 0.05	-6.03 ± 0.06		
G79I	11.90 ± 0.19	3.46 ± 0.06	12.96 ± 0.16	3.60 ± 0.05		
G79V	9.87 ± 0.12	3.74 ± 0.06	11.50 ± 0.18	3.95 ± 0.06		
G79L	5.45 ± 0.43	1.51 ± 0.07	5.64 ± 0.25	1.57 ± 0.06		
G79A	5.07 ± 0.05	2.16 ± 0.04	6.33 ± 0.11	2.10 ± 0.04	4.93 ± 0.04	4.82 ± 0.03
V80I	-0.45 ± 0.05	-0.32 ± 0.04	-0.45 ± 0.05	-0.56 ± 0.05		
V80L	-7.17 ± 0.07	-3.78 ± 0.04	-7.41 ± 0.06	-3.80 ± 0.06		
V80A	-4.97 ± 0.03	-2.81 ± 0.05	-4.79 ± 0.05	-2.74 ± 0.04		
V80G	-4.07 ± 0.08	-2.48 ± 0.05	-3.99 ± 0.07	-2.61 ± 0.06		
V80T	-10.68 ± 0.06	-5.65 ± 0.12	-10.83 ± 0.05	-5.68 ± 0.06		
V80S	-13.13 ± 0.05	-7.19 ± 0.08	-13.13 ± 0.05	-7.06 ± 0.09		
G83I	12.27 ± 0.15	3.67 ± 0.05	12.16 ± 0.27	3.68 ± 0.05	9.16 ± 0.16	9.87 ± 0.07
G83V	10.42 ± 0.22	3.62 ± 0.05	10.25 ± 0.08	3.14 ± 0.05		
G83L	9.05 ± 0.27	1.51 ± 0.05	9.51 ± 0.21	1.19 ± 0.05		
G83A	5.62 ± 0.05	2.13 ± 0.03	4.64 ± 0.03	2.05 ± 0.02	3.69 ± 0.02	2.94 ± 0.03
G83S	-1.81 ± 0.21	-1.91 ± 0.04	-0.80 ± 0.06	-2.04 ± 0.05		
G83T	-0.16 ± 0.12	-1.44 ± 0.04	-1.35 ± 0.11	-1.29 ± 0.05	-1.49 ± 0.08	-2.02 ± 0.06
V84I	-0.80 ± 0.08	-0.52 ± 0.04	-0.58 ± 0.05	-0.55 ± 0.04		
V84L	-8.49 ± 0.11	-4.15 ± 0.05	-8.14 ± 0.08	-4.28 ± 0.05	-8.77 ± 0.08	-8.18 ± 0.04
V84A	-7.20 ± 0.05	-2.51 ± 0.05	-7.19 ± 0.04	-2.57 ± 0.03	-7.67 ± 0.05	-6.86 ± 0.04
V84G	-7.26 ± 0.05	-2.71 ± 0.05	-7.00 ± 0.05	-2.70 ± 0.05	-6.76 ± 0.08	-6.97 ± 0.06
V84T	-12.19 ± 0.05	-5.96 ± 0.08	-10.74 ± 0.06	-5.45 ± 0.04		
V84S	-18.52 ± 0.09	-6.99 ± 0.06	-18.32 ± 0.08	-6.95 ± 0.07		
T87I	10.54 ± 0.07	4.97 ± 0.04	9.21 ± 0.09	3.99 ± 0.05	10.67 ± 0.13	10.83 ± 0.08
T87V	16.61 ± 0.13	5.69 ± 0.04	12.61 ± 0.08	4.56 ± 0.04		
T87L	2.87 ± 0.09	0.74 ± 0.04	2.74 ± 0.07	0.88 ± 0.05	2.49 ± 0.80	2.76 ± 0.07
T87A	4.68 ± 0.06	2.82 ± 0.03	4.94 ± 0.05	2.99 ± 0.02	5.23 ± 0.05	5.42 ± 0.05
T87G	2.92 ± 0.06	1.96 ± 0.03	2.59 ± 0.09	1.94 ± 0.04	2.30 ± 0.09	3.33 ± 0.09
T87S	-3.50 ± 0.09	-1.48 ± 0.05	-3.90 ± 0.09	-1.22 ± 0.06	-4.49 ± 0.10	-4.09 ± 0.11

Table S2: Alchemical free energy change of monomer in PLPC and PYPC bilayer and in water, given in kcal/mol.

Mutation \ Enviroment	PYPC	PLPC	Water
L75I	3.89 ± 0.05	4.47 ± 0.06	3.61 ± 0.08
L75V	4.49 ± 0.04	3.79 ± 0.10	3.57 ± 0.08
L75A	1.40 ± 0.05	1.48 ± 0.06	-0.41 ± 0.07
L75G	-4.42 ± 0.06	-4.97 ± 0.27	-3.45 ± 0.08
I76V	0.68 ± 0.05	0.35 ± 0.04	0.01 ± 0.04
I76L	-3.90 ± 0.12	-3.92 ± 0.11	-3.99 ± 0.09
I76A	-2.95 ± 0.05	-1.35 ± 0.03	-3.74 ± 0.07
I76G	-3.68 ± 0.06	-3.64 ± 0.27	-5.24 ± 0.08
I76S	-8.08 ± 0.07	-8.06 ± 0.07	-10.27 ± 0.09
G79I	3.84 ± 0.07	3.62 ± 0.06	5.25 ± 0.08
G79V	3.71 ± 0.05	4.07 ± 0.06	7.01 ± 0.08
G79L	1.59 ± 0.08	1.35 ± 0.08	3.45 ± 0.08
G79A	2.14 ± 0.04	2.33 ± 0.28	-3.58 ± 0.06
V80I	-0.38 ± 0.07	-0.18 ± 0.11	-0.18 ± 0.08
V80L	-4.06 ± 0.06	-3.50 ± 0.10	-3.69 ± 0.07
V80A	-2.73 ± 0.08	-2.49 ± 0.08	-3.59 ± 0.06
V80G	-3.50 ± 0.05	-2.64 ± 0.05	-4.69 ± 0.06
V80T	-5.42 ± 0.12	-5.10 ± 0.12	-8.09 ± 0.08
V80S	-6.51 ± 0.11	-6.22 ± 0.09	-10.29 ± 0.07
G83I	3.84 ± 0.06	3.79 ± 0.05	6.37 ± 0.08
G83V	3.25 ± 0.05	3.34 ± 0.05	5.38 ± 0.07
G83L	1.16 ± 0.05	1.35 ± 0.05	3.93 ± 0.08
G83A	2.14 ± 0.03	2.36 ± 0.22	3.06 ± 0.04
G83T	-1.34 ± 0.05	-1.39 ± 0.04	-2.32 ± 0.07
G83S	-1.90 ± 0.05	-2.07 ± 0.05	-1.90 ± 0.05
V84I	0.07 ± 0.09	-0.39 ± 0.09	-0.19 ± 0.05
V84L	-3.80 ± 0.08	-4.36 ± 0.09	-3.76 ± 0.04
V84A	-2.59 ± 0.03	-2.82 ± 0.07	-3.55 ± 0.06
V84G	-2.61 ± 0.04	-2.92 ± 0.06	-4.54 ± 0.04
V84T	-5.31 ± 0.09	-5.11 ± 0.10	-8.10 ± 0.05
V84S	-6.90 ± 0.09	-5.89 ± 0.10	-10.09 ± 0.07
T87I	5.04 ± 0.04	5.35 ± 0.04	8.87 ± 0.09
T87V	5.53 ± 0.04	5.63 ± 0.13	8.70 ± 0.09
T87L	0.88 ± 0.11	1.11 ± 0.04	4.39 ± 0.09
T87A	2.71 ± 0.03	3.15 ± 0.10	4.95 ± 0.07
T87G	1.88 ± 0.04	1.93 ± 0.04	3.02 ± 0.07
T87S	-1.41 ± 0.07	-1.46 ± 0.06	-2.33 ± 0.09

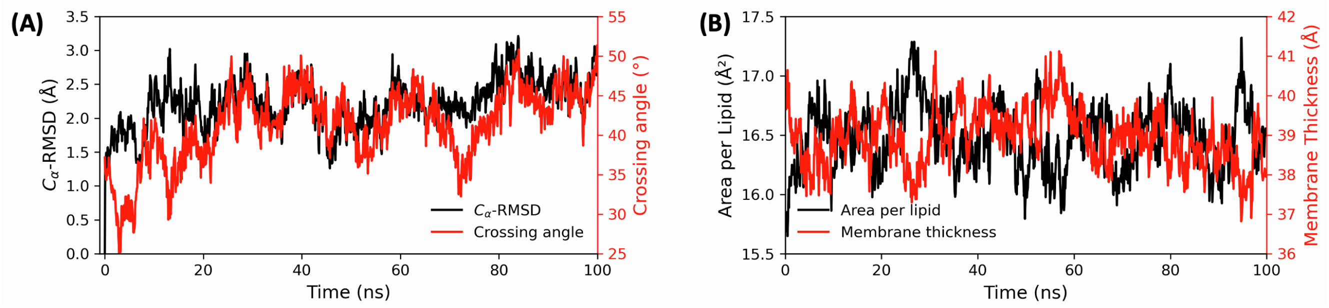

Figure S1: **Equilibration simulation of wildtype GpA-dimer in POPC membrane**. Data for other membranes and mutants have a similar fluctuation and are not shown. **(A)** RMSD relative to the first frame and crossing angle. **(B)** Membrane thickness and area per lipid.

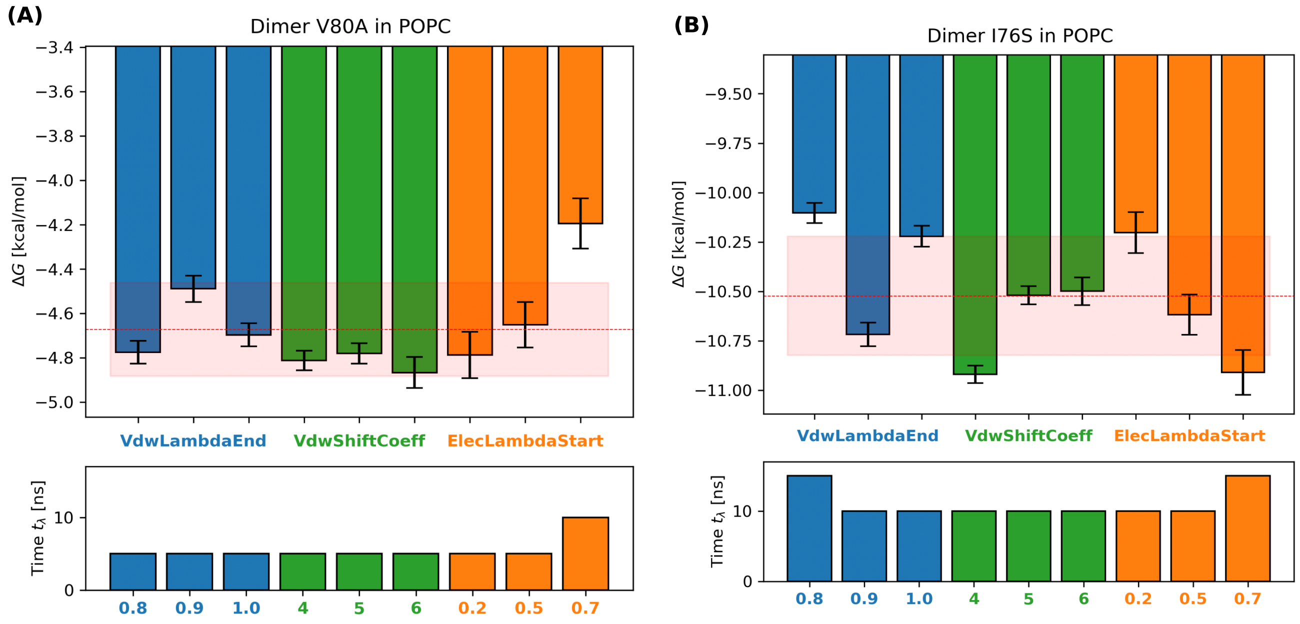

Figure S2: **Setup optimization**. Free energy changes ΔG were tested against the alchemical non-bonded parameters implemented in the NAMD (42) for small and large residue transformations of dimer V80A and I76S in POPC membrane, respectively. The red line is the average of ΔG of all parameters, with the shaded region presenting its SEM.

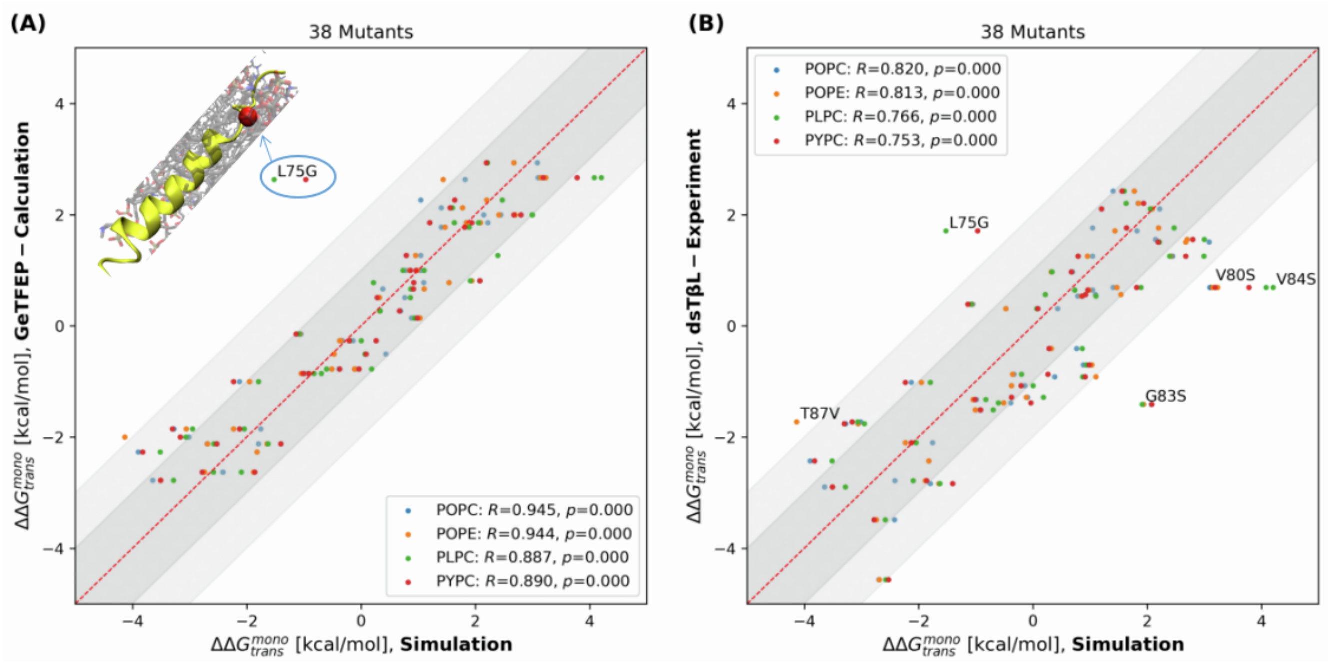

Figure S3: Correlation of simulated relative transfer free energy changes of the monomer $\Delta\Delta G_{trans}^{mono}$ in different lipid membranes with: **(A)** the theoretical predictions from GeTFEP (56) and **(B)** experiments dsTβL (6). The correlation coefficient and the statistical significance are denoted with R and p, respectively. The shaded regions represent a free energy change range of $1 - 2\,\mathrm{kcal/mol}$.

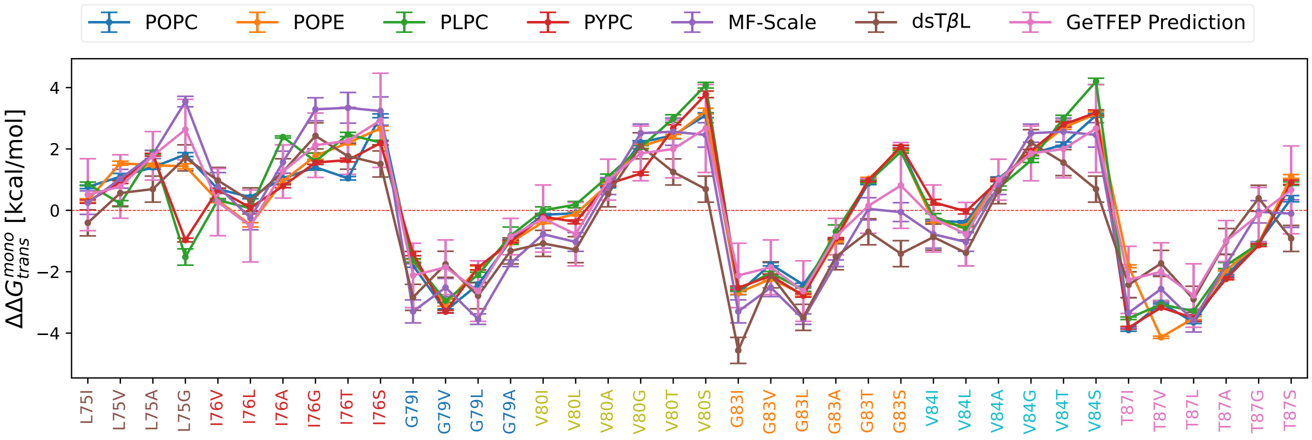

Figure S4: Full data presentation as supplement of Fig. 3. In addition, the theoretical prediction GeTFEP (56) is presented.

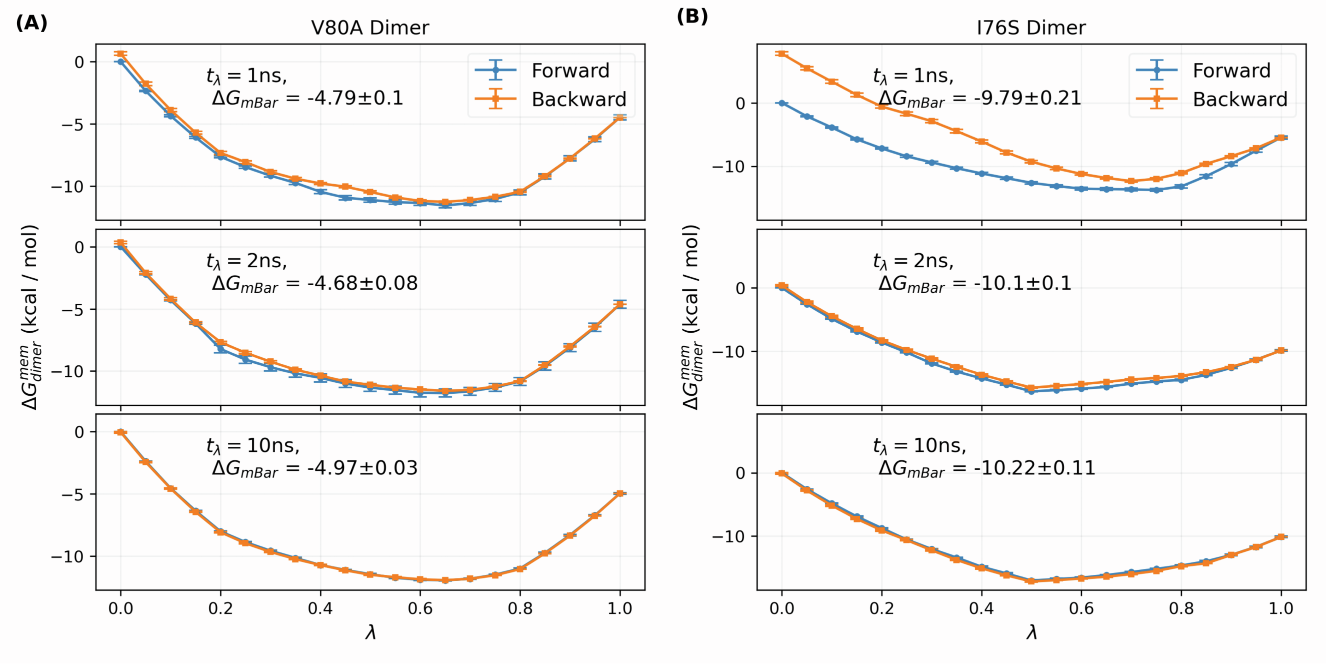

Figure S5: Free energy convergence analysis based on forward and backward transformations for example test cases **(A)** V80A dimer and **(B)** I76S dimer. The simulated membrane is POPC, and ΔG_{mBar} presents the final free energy change estimated by the mBAR method (31).

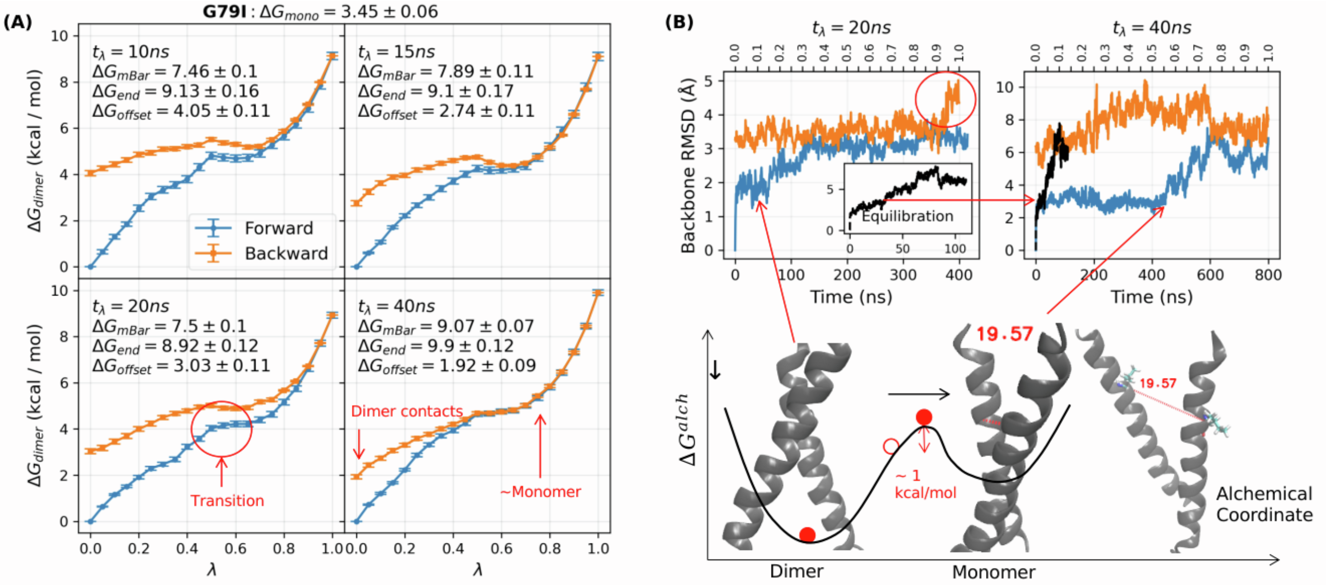

Figure S6: **Observable dimer distortion during alchemical transformation of G79I mutant**. **(A)** Alchemical free energy profile of forward and backward simulation with increasing sampling times t_λ. The ΔG_{mbar}, ΔG_{end}, and ΔG_{offset} are the free energy change approximated by the mBar method (31), extracted by the end of the forward and backward simulation, respectively. The free energy change of the G79I monomer mutant ΔG_{mono} is given for comparison. **(b)** RMSD of the dimer during the alchemical transformation corresponding to t_λ in **(A)**. The conformational dynamics during the forward transformation are visualized with an illustrated energy landscape (black curve), indicating a dimer-monomer transition tendency, from which an energetic barrier of 1 kcal/mol may be estimated. The distance between the mutated residue G79I is denoted with a red connection line. The inset shows the RMSD of the separate 100 ns equilibrium of the G79I mutant (black curve) prepared by CHARMM-GUI (37). The red ball denotes the transition state upon increased sampling.

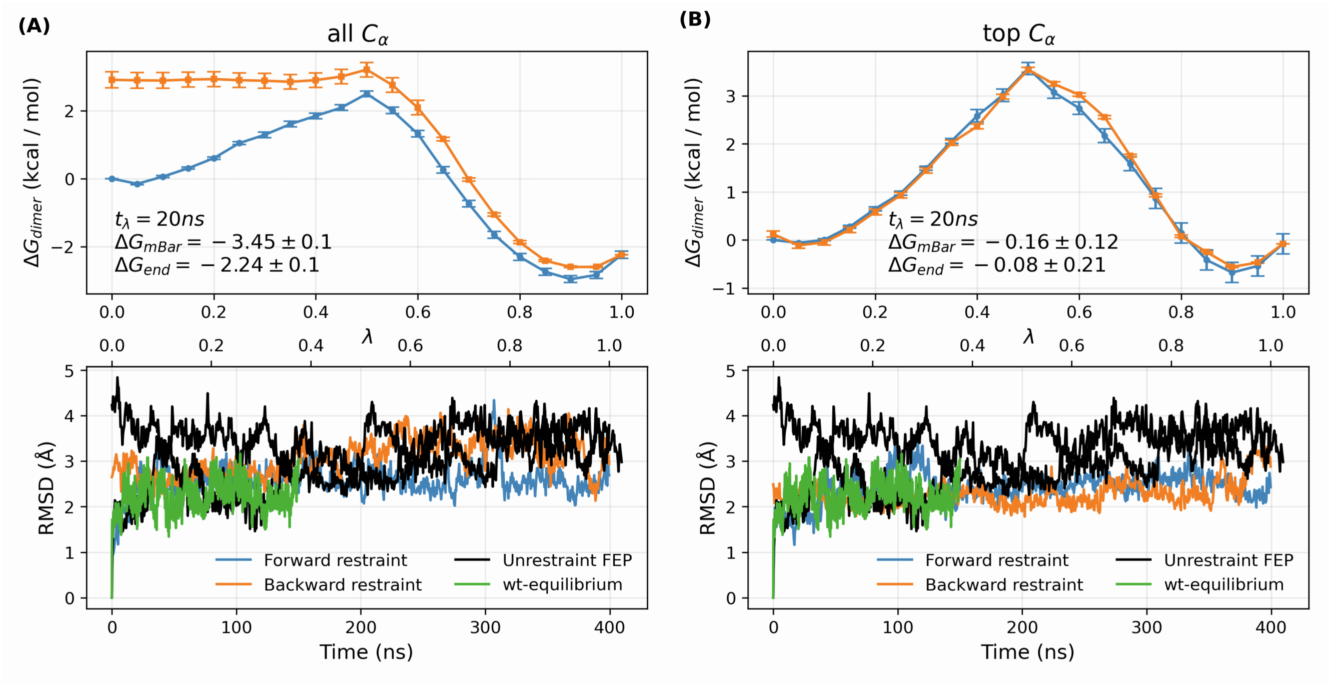

Figure S7: **Approach to Solving the Convergence Problem of GxxxG Mutations: Case Study of the G83T Dimer Mutant**. Here, the positional restraint using a weak force constant of 0.1 kcal/mol/$\mathring{A}^2$ was applied only during the pre-equilibrium phase before initiating the alchemical transformation. The restraint was then released for the alchemical simulation in two scenarios: (**A**) for all C_α and (**B**) for the four top $C_{\alpha,96,77}$ of the dimer.

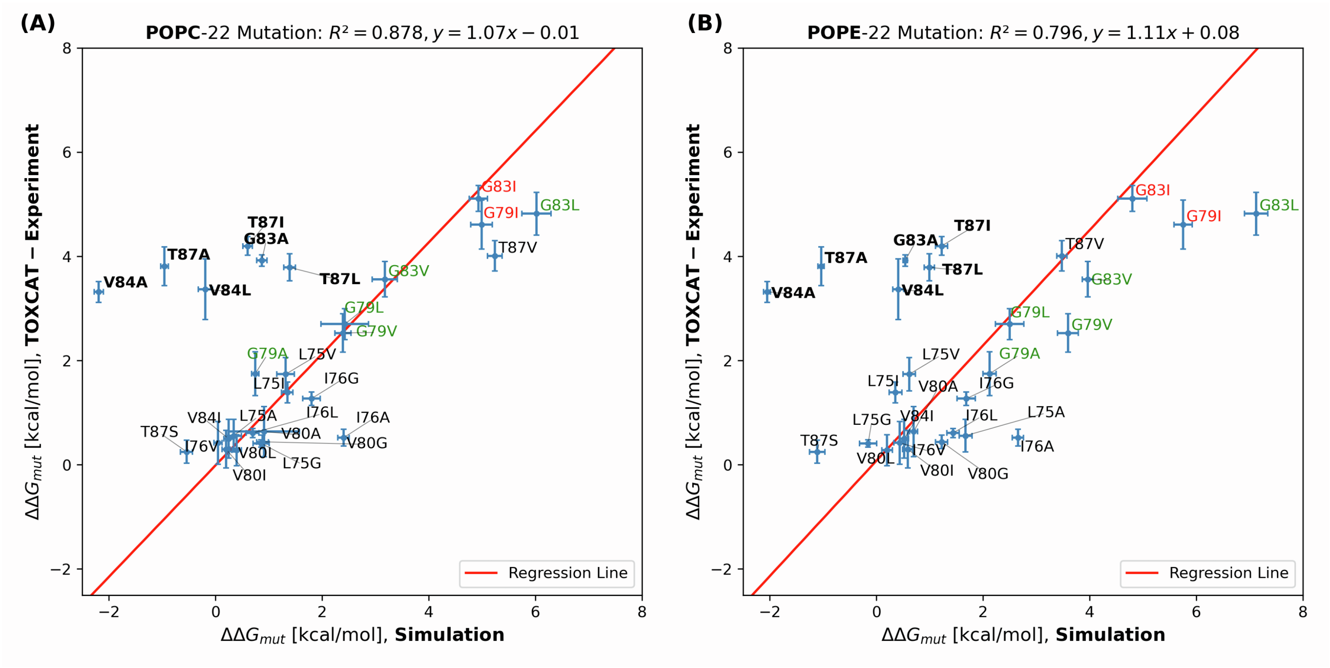

Figure S8: Linear regression of relative dimerization free energy changes between TOXCAT experiment (21) and simulation in (**A**) POPC and (**B**) POPE membrane. The correlation coefficient R^2 and the regression line p were calculated after excluding the seven outlier mutations (bold printed). For the mutated dimer with a drastic conformation change, mutations that required positional restraints for convergence are indicated in green, while those with energy estimates based solely on single forward simulations are shown in red.

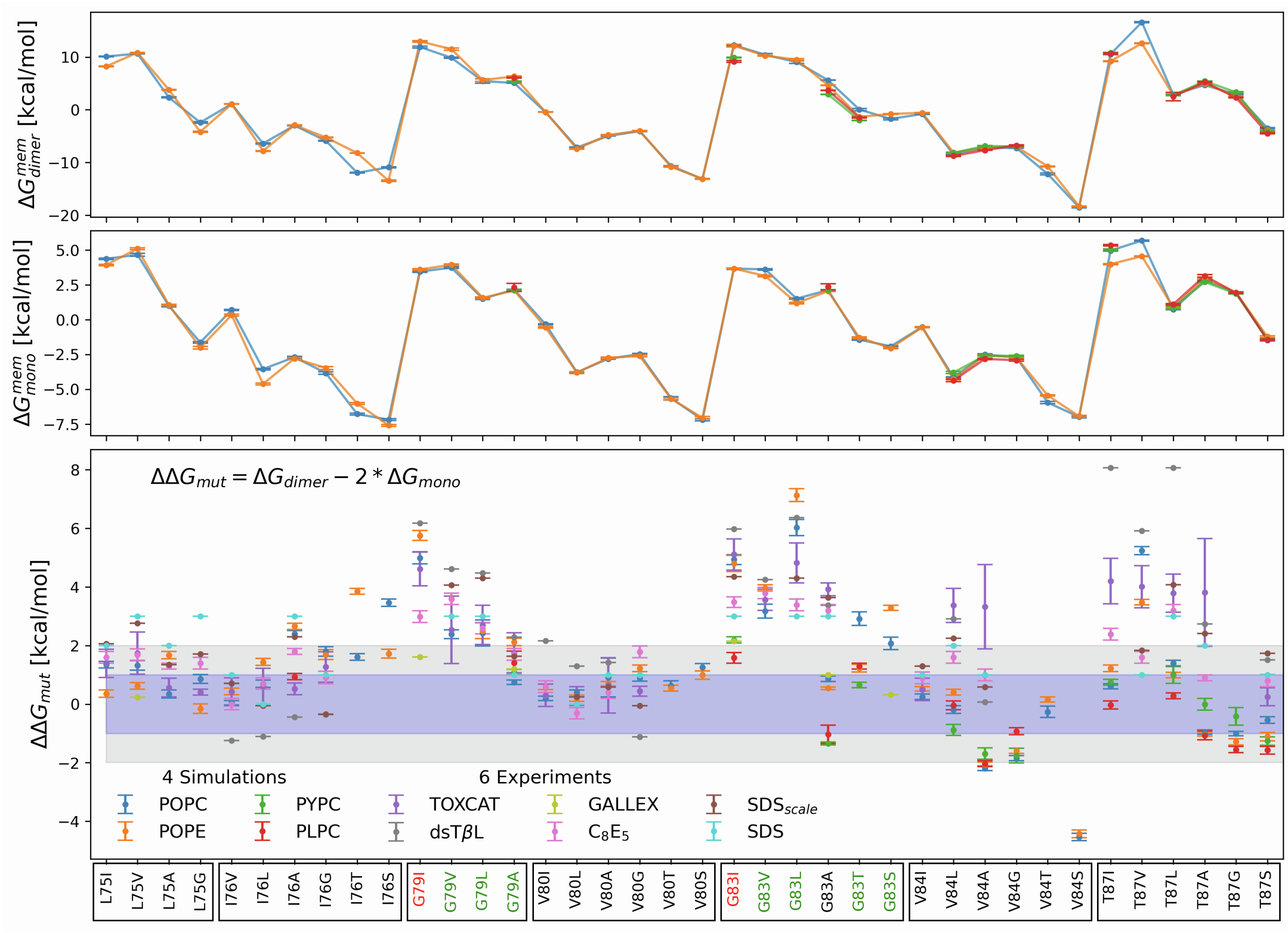

Figure S9: Full presentation of calculated alchemical free energy change of dimer (ΔG_{dimer}^{mem}) and monomer (ΔG_{mono}^{mem}) upon single point mutation in different lipid environments, resulting in the relative change between mutants and wild-type $\Delta\Delta G_{mut}$.

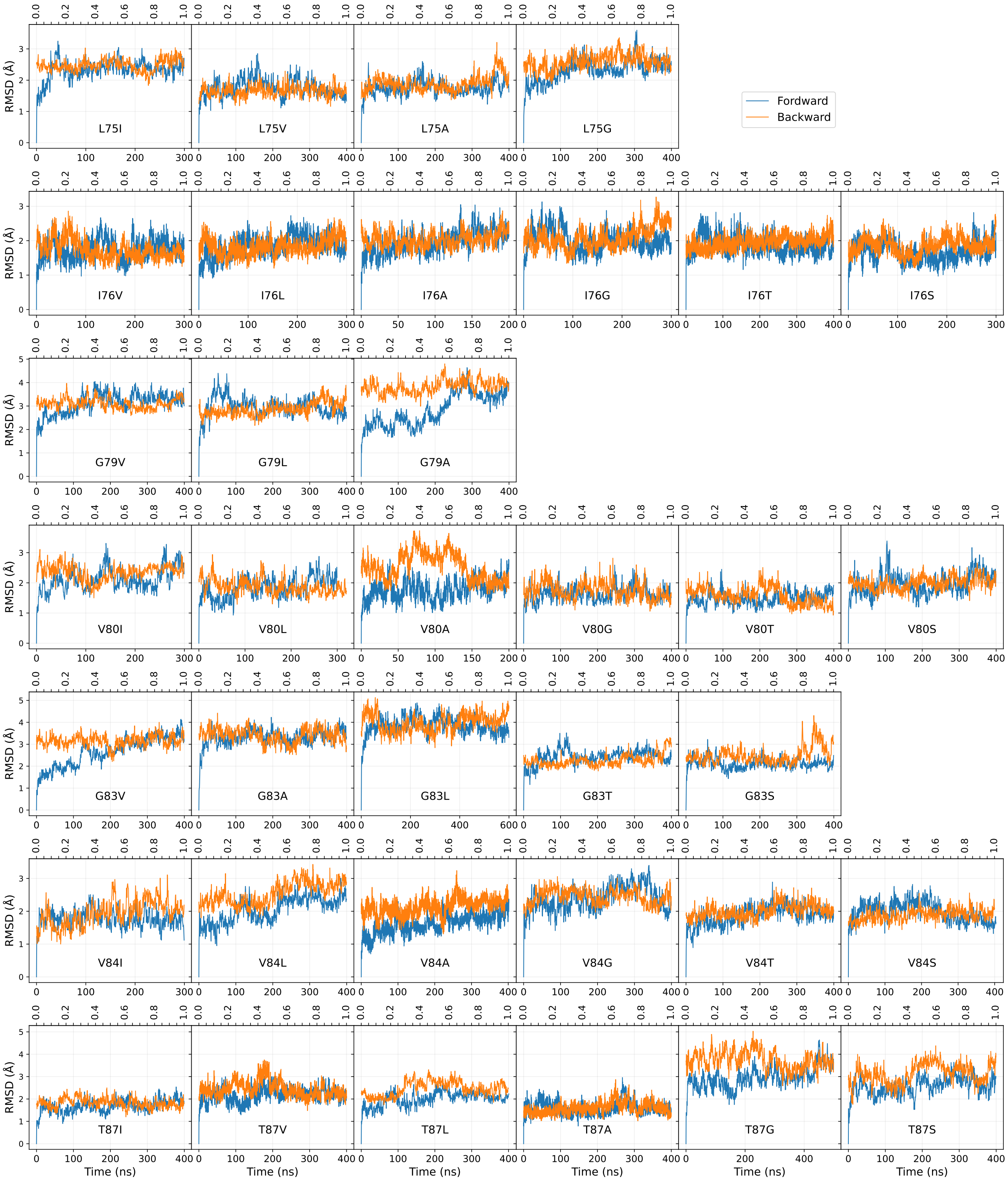

Figure S10: **GpA-dimer in POPC bilayer membrane**: Backbone RMSD calculated from the FEP trajectories with the longest sampling time for both forward and backward simulation. Data for the POPE bilayer is not shown since it is similar.

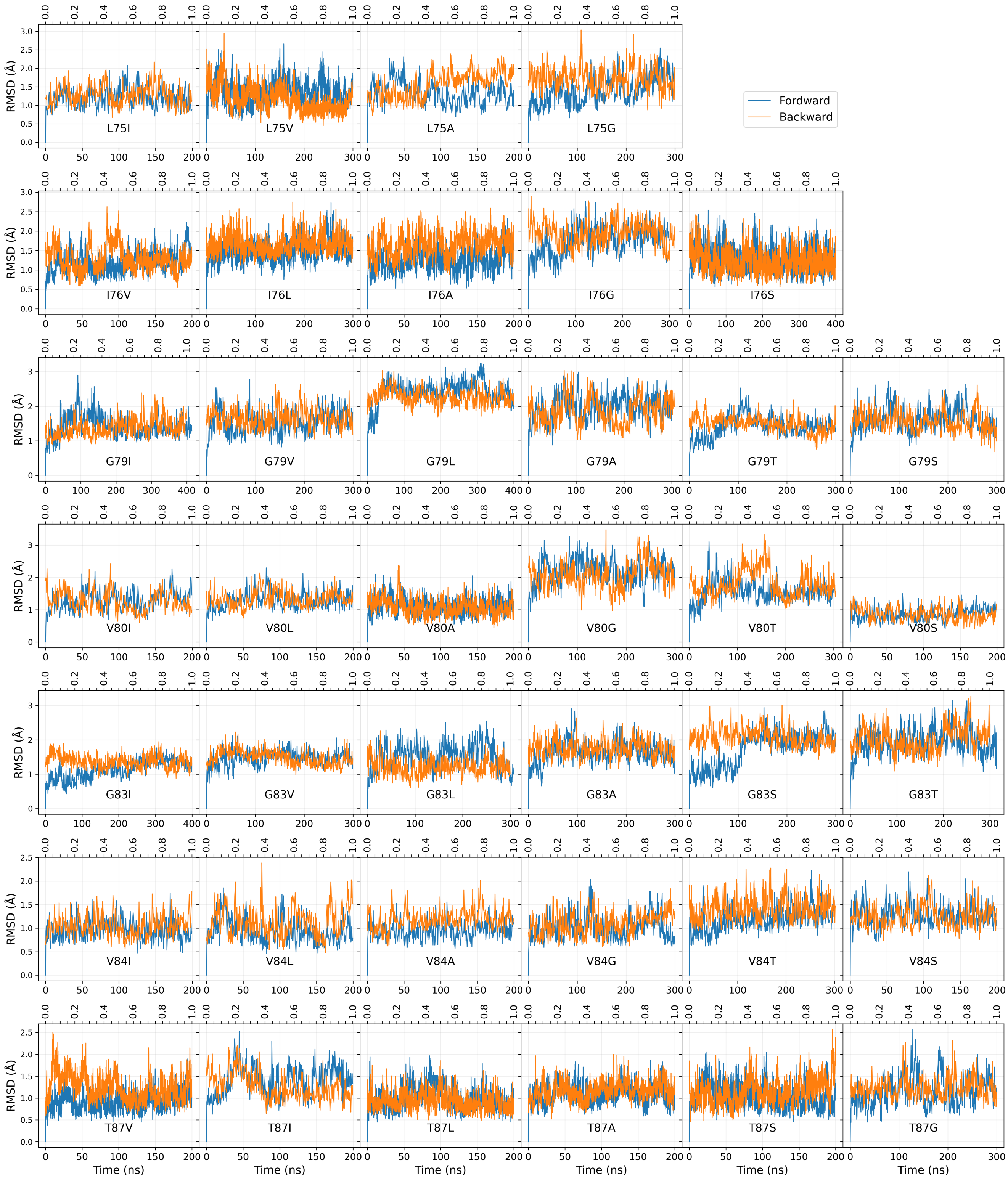

Figure S11: **GpA-monomer in POPC bilayer membrane**: Backbone RMSD calculated from the FEP trajectories with the longest sampling time for both forward and backward simulation. Data for the POPE bilayer is not shown since it is similar.

B MD SIMULATION SUPPLEMENT

To complement the free energy calculations and focus on the protein-protein interactions, we performed separate 100 ns molecular dynamics simulations of the mutants in a POPC bilayer, using initial structures prepared with CHARMM-GUI (37). Averages were computed over the final 50 ns of the equilibrated trajectories, as shown in the representative example in Fig. S1.

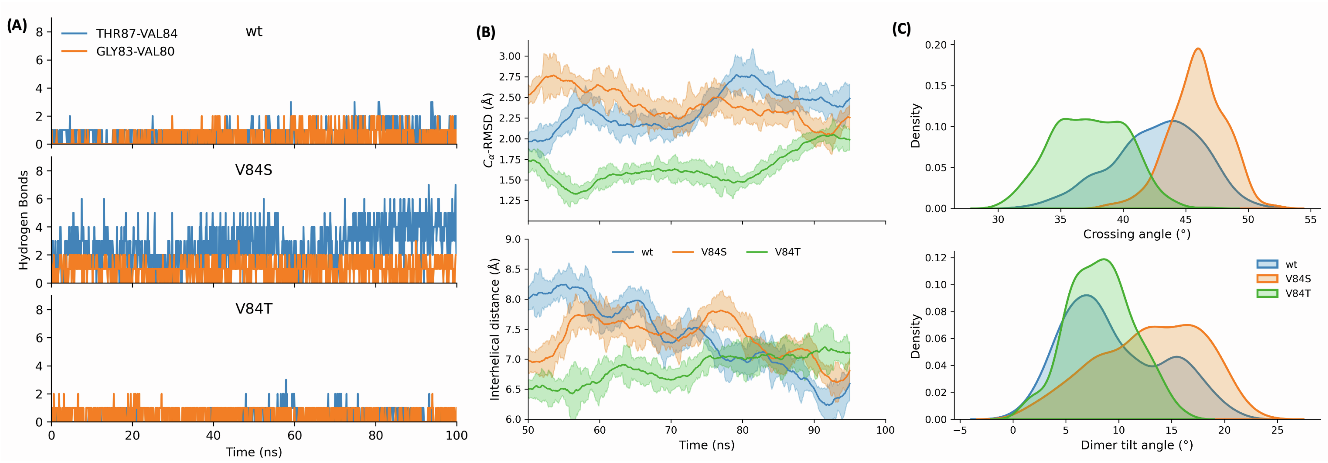

Figure S12: Equilibrium simulation of V84S and V84T mutants compared to the wildtype in POPC bilayer. **(A)** Hydrogen bond count between selected residues from each frame, presented as accumulation in each time window. **(B)** Dynamics of backbone RMSD and the center of mass interhelical distance. **(C)** Density distribution of dimer crossing and tilt angle. The tilt angle is defined as the angle between the membrane normal and the vector sum of the helical axes of the two monomers, while the crossing angle is the angle between the two helical axes.

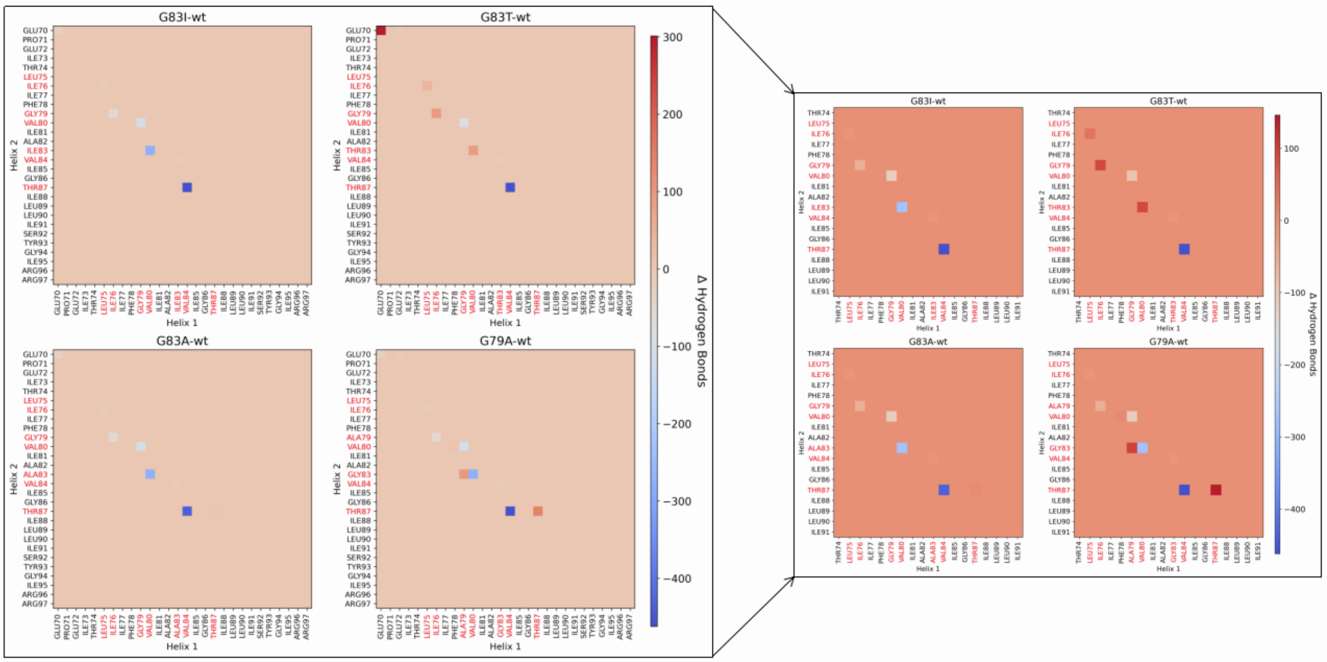

Figure S13: Residue-wise heatmap showing differences in interhelical hydrogen bonding between the GpA dimer and its mutants in a POPC bilayer. The total number of hydrogen bonds involving the C_α atoms of each residue pair across the two helices was averaged over the final 50 ns of the equilibrated trajectories. The heatmap depicts values obtained by subtracting the mutant interhelical hydrogen-bond counts from those of the wild type (wt). The inset provides a zoomed-in view of the interface residue region.

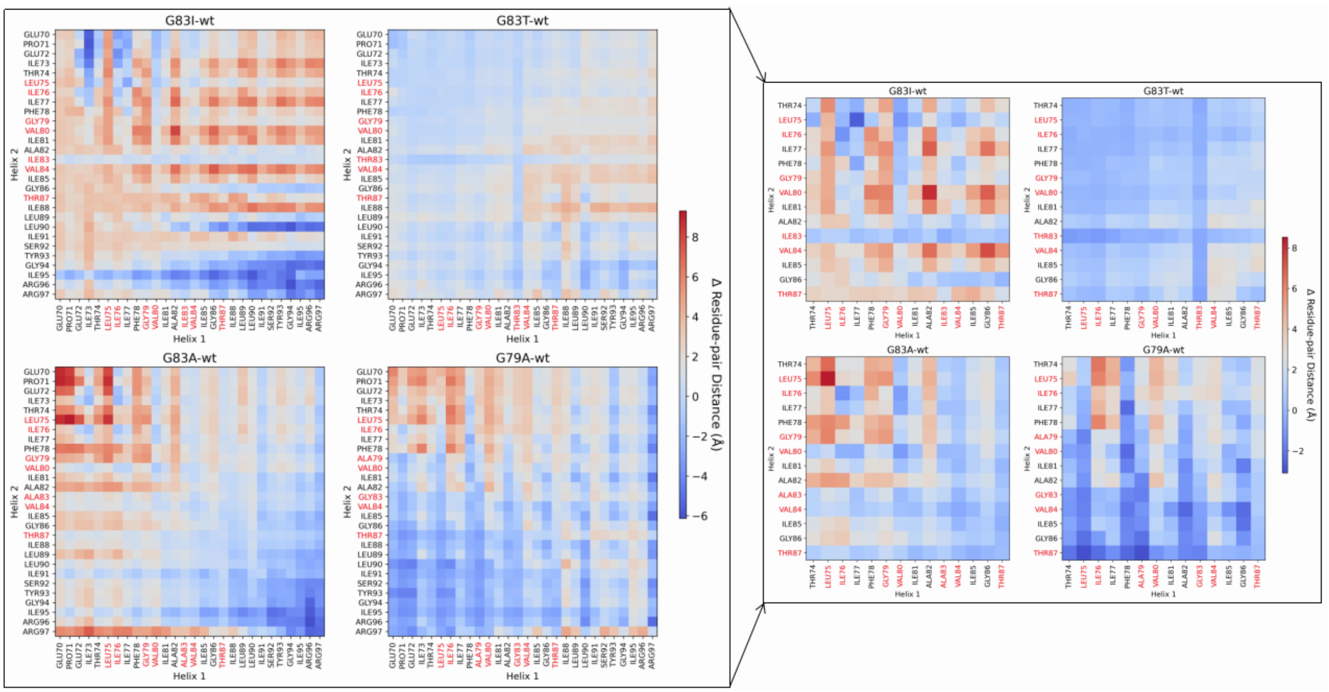

Figure S14: Residue-wise heatmap of interhelical distance differences between the GpA dimer and its GxxxG mutant in a POPC bilayer. The distance between the C_α atoms of each residue on the two helices was averaged over the final 50 ns of the equilibrated trajectory. The heatmap depicts values obtained by subtracting the mutant interhelical distance from those of the wild type (wt). The inset provides a zoomed-in view of the interface residue region.

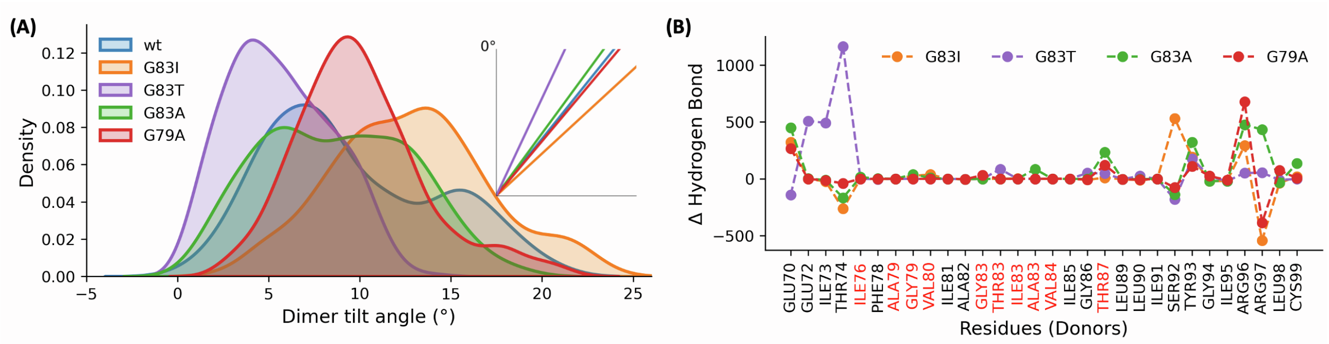

Figure S15: Structural and thermodynamic characterization of GpA dimer mutations in POPC bilayer compared to the wild type (wt). **(A)** Density distribution of dimer tilt angle with the inset showing the maximal values as angle relative to the membrane normal. **(B)** Difference in protein–lipid hydrogen bond counts between wt and mutants, with lipids as hydrogen bond acceptors and protein residues as donors.
